# Impulsive-compulsive behaviours and striatal neuroactivity in mildly parkinsonian rats under D2/3 agonist and L-DOPA treatment

**DOI:** 10.1038/s41531-025-00996-z

**Published:** 2025-05-29

**Authors:** Mirjam Wolfschlag, Elena Espa, Katrine Skovgård, Pär Halje, Maria Angela Cenci

**Affiliations:** 1 Basal Ganglia Pathophysiology Unit, Department of Experimental Medical Science, Lund University, Lund, Sweden; 2 Department of Clinical Sciences Lund, Psychiatry, Faculty of Medicine, Lund University, Lund, Sweden; 3 Malmö Addiction Center, Clinical Research Unit, Malmö, Sweden; 4 The Group for Integrative Neurophysiology and Neurotechnology, Department of Experimental Medical Science, Lund University, Lund, Sweden

**Keywords:** Basal ganglia, Parkinson's disease, Clinical pharmacology

## Abstract

Dopamine replacement therapy for Parkinson’s disease can induce impulsive-compulsive behaviours (ICBs). Here we compare the D2/3 agonist ropinirole and L-DOPA, given alone or combined, with regard to their potential to induce ICBs in rats sustaining bilateral striatal injections of 6-hydroxydopamine. Daily treatment with ropinirole (2.5 mg/kg), L-DOPA (24.0 mg/kg), or their combination was given for six weeks while animals were examined using tests of compulsive checking and motor stereotypies not previously used in the ICB literature. Independently of L-DOPA cotreatment, ropinirole induced a stereotyped hyperactivity pattern, compulsive checking, and maladaptive choices in the rat version of the Iowa gambling task. Compared to both L-DOPA and vehicle, ropinirole elicited a distinct pattern of striatal neuroactivity, shifting the expression of a cellular activity marker from dorsolateral to centro-medial regions. Our results reveal quite distinct profiles of ICBs and striatal activation upon treatment with ropinirole or L-DOPA, providing clues of therapeutic relevance to Parkinson’s ICBs.

## Introduction

Parkinson’s disease (PD) is characterised by a severe degeneration of dopaminergic neurons in the substantia nigra pars compacta with an ensuing depletion of dopamine (DA) in the striatum. Parkinsonian motor symptoms are alleviated by the DA precursor L-DOPA, which causes motor fluctuations and dyskinesia in the vast majority of PD patients^1–3^. The use of L-DOPA is often delayed in favour of DA agonists that directly stimulate DA receptors. Although often used as a monotherapy in early-stage PD, DA agonists are generally combined with L-DOPA for improved symptomatic control as the disease becomes more severe^1,4^.

The use of DA agonists is associated with an increased risk of developing impulsive-compulsive behaviours (ICBs), which are particularly frequent upon treatment with the high-affinity D2/3 receptor agonists pramipexole and ropinirole^5,6^. ICBs are characterised by an inability to exert self-control emotionally and operantly, which can result in maladaptive choices and actions^7^. Impulsivity has been related to risk-taking, lack of mental planning, and rapid decision-making^8^, while compulsivity is characterised by repetitive, perseverative, non-goal-oriented behaviours^7,9^. Several large-scale studies in PD patients have shown that ICB can manifest as pathological gambling, compulsive sexuality or buying, and binge eating^10–14^. Additionally, repetitive, purposeless behaviours called punding have been recognised as a side effect of dopaminergic medication in PD^5,15^.

Research in animal models of PD treated with DA agonists has grown as an important translational area to elucidate the neurobiological basis of ICBs and identify therapeutic targets^5,16–18^. There is now excellent consensus that rats treated with D2/3 agonists exhibit ICB-like features which are typically assessed using operant tests of impulsive decision-making^19–24^. Despite being frequently used in the clinic, regimens of DA agonist and L-DOPA coadministration have not yet been evaluated in any animal model of ICBs.

In this study, we set out to compare the effects of the D2/3 agonist ropinirole, alone or combined with L-DOPA, on behavioural measures of ICBs. As a model, we used rats with bilateral dopaminergic denervation induced by 6-hydroxydopamine (6-OHDA) restricted to the dorsolateral striatum, which are commonly used in experimental studies of PD therapy-induced ICBs^20,22–26^. Behavioural outcomes were assessed using non-operant tasks, circumventing the need for lengthy training periods^16^, and thus suitable to be applied sequentially within a drug treatment period of six weeks. Compulsive features were extracted from the analysis of behavioural states in freely moving animals and moreover evaluated using a test of compulsive checking originally developed to study obsessive-compulsive disorders^27^, here applied to a PD model for the first time. Risk taking behaviour and disadvantageous decision making, typical for pathological gambling, were modelled using the rat analogue of the Iowa Gambling Task (rIGT)^28^, developed to assess gambling behaviour in patients^29^.

At the end of the behavioural studies, the rat brains were used to assess the patterns of striatal DA denervation and the cellular expression of phosphorylated ribosomal protein S6 (pS6), a neuronal activity marker^30,31^. Our results highlight the neuropsychiatric liability associated with D2/3 agonist treatment and reveal different effect profiles for ropinirole and L-DOPA on behavioural and cellular activity patterns in the rat.

## Results

### Evaluation of the 6-OHDA lesion and motor effects of the treatments

**Fig. 1:**
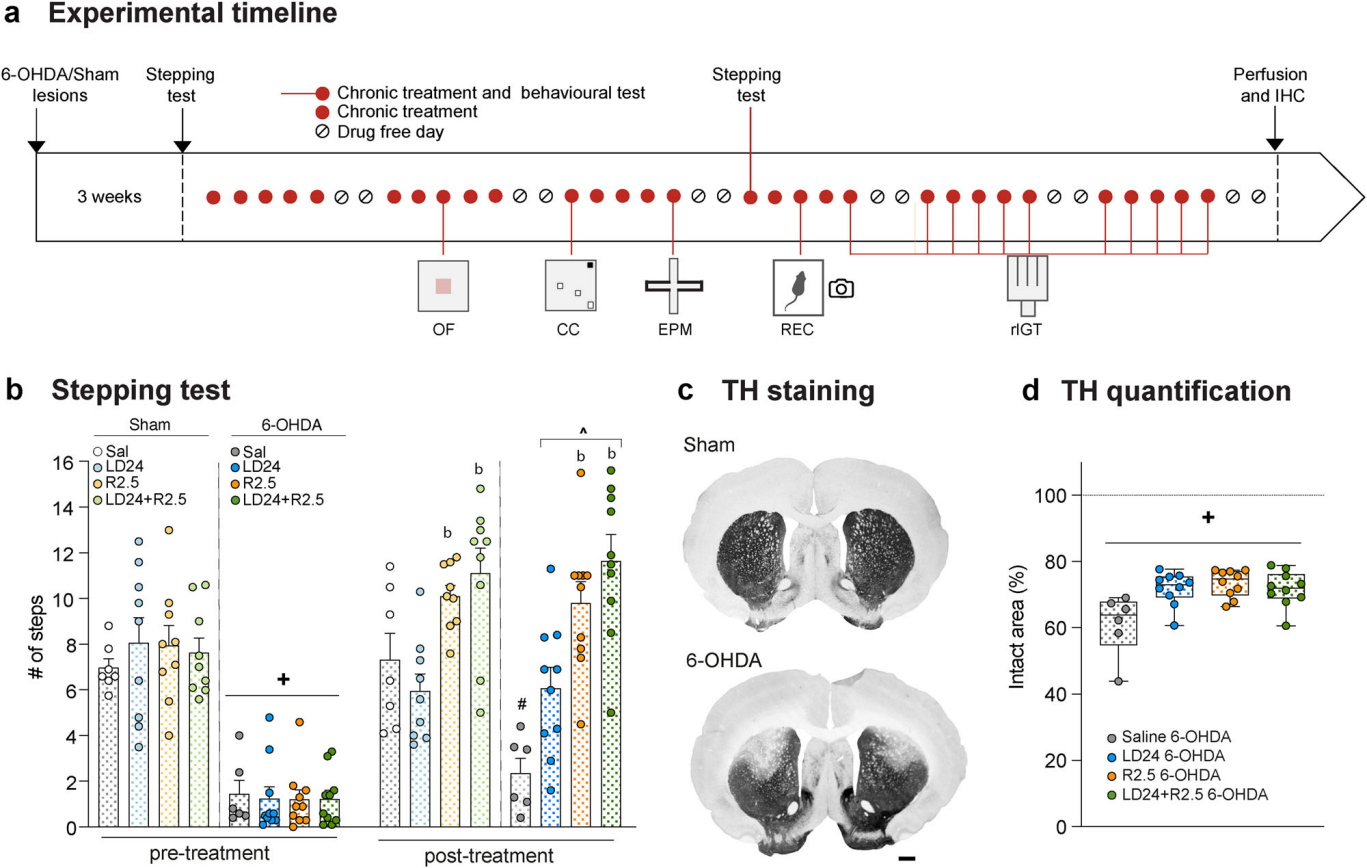
Study design and validation of 6-hydroxydopamine lesions. **a** Schematic representation of the study design. Rats were injected bilaterally with the toxin 6-hydroxydopamine (6-OHDA) in the dorsolateral striatum (*n* = 36), and a separate group of rats sustained bilateral sham lesions (*n* = 34). Both groups received treatment with L-DOPA 24.0 mg/kg (LD24), ropinirole 2.5 mg/kg (R2.5), or the combination of L-DOPA and ropinirole (LD24 + R2.5). An additional group of sham- and 6-OHDA-lesioned animals received vehicle treatment (0.9% saline) throughout the experiment. Rats were tested in the open field test (OF, week 2 of chronic treatment), in the compulsive checking test (CC, week 3), and in the elevated plus maze (EPM, week 3). Then, the video recording of active behaviours (REC, week 4) was performed before the rat Iowa Gambling Task (rIGT, week 4–6). **b** Stepping test performance before treatment (pre-treatment; left) and in week 4 of chronic treatment (post-treatment; right). (Repeated measurement mixed effect model: F(treatment)_7,36_ = 19.6, *p* < 0.001; F(time)_1,9_ = 75.0, *p* < 0.001; F(interaction)_7,43_ = 15.8, *p* < 0.001). **c** Representative sections of the striatum stained for tyrosine hydroxylase (TH) in bilaterally sham- (top) or 6-OHDA-lesioned rats (bottom). Scale bar: 600 µm. **d** TH quantification was performed in the dorsal striatum. Data are expressed as % of intact area and the dashed line marks the average % intact area in sham-lesioned animals (Kruskal-Wallis test: KW(treatment)=28.3, *p* < 0.001). Symbols of statistical significance: + = *p* < 0.05 vs all Sham pre-treatment; # = *p* < 0.05 vs all 6-OHDA groups post-treatment; b = *p* < 0.05 vs LD24 of same lesion type; ^ = *p* < 0.05 vs. same lesion and treatment (pre).

Bilateral 6-OHDA- or sham-lesioned rats received treatment with saline, L-DOPA 24.0 mg/kg (LD24), ropinirole 2.5 mg/kg (R2.5) or the combination of L-DOPA and ropinirole (LD24 + R2.5), according to the study design illustrated in Fig. 1a. To verify the efficacy of striatal DA denervation, animals were evaluated on a test of forelimb akinesia (stepping test)^32^. As expected, infusion of 6-OHDA in the dorsolateral striatum resulted in a reduced number of forelimb adjusting steps (Fig. 1b; *p* < 0.05 for 6-OHDA vs sham groups), and no significant difference was found at baseline between animals allocated to the different treatment groups (Fig. 1b, see pre-treatment graph). Importantly, each of the drug treatments under investigation significantly improved this motor impairment (Fig. 1b, post-treatment graph, *p* < 0.05 vs pre-treatment values in each 6-OHDA group treated with R2.5, LD24, or LD24 + R2.5; non-significant difference vs the sham groups). Incidentally, treatment with ropinirole alone or combined with L-DOPA resulted in a larger number of adjusting steps compared with L-DOPA alone (Fig. 1b; *p* < 0.05 for R2.5 and LD24 + R2.5 vs LD24 treatment in both sham and 6-OHDA cohorts; *p* < 0.05 for 6-OHDA saline vs all other groups).

At the end of the behavioural studies, the extent of striatal dopaminergic denervation was verified using tyrosine hydroxylase (TH) immunohistochemistry. In keeping with the behavioural results, all lesioned animals displayed partial TH loss in the dorsolateral striatum (Fig. 1c, d) and a reduced number of TH-immunoreactive cells in the substantia nigra pars compacta (Fig. S5b). On either parameter, no significant difference in lesion extent was found between the different treatment groups (Figs. 1d, S5b; *p* < 0.05 vs Sham for each treated group in the 6-OHDA cohort).

### Ropinirole, but not L-DOPA, induced hyperactivity with signs of reduced anxiety

**Fig. 2:**
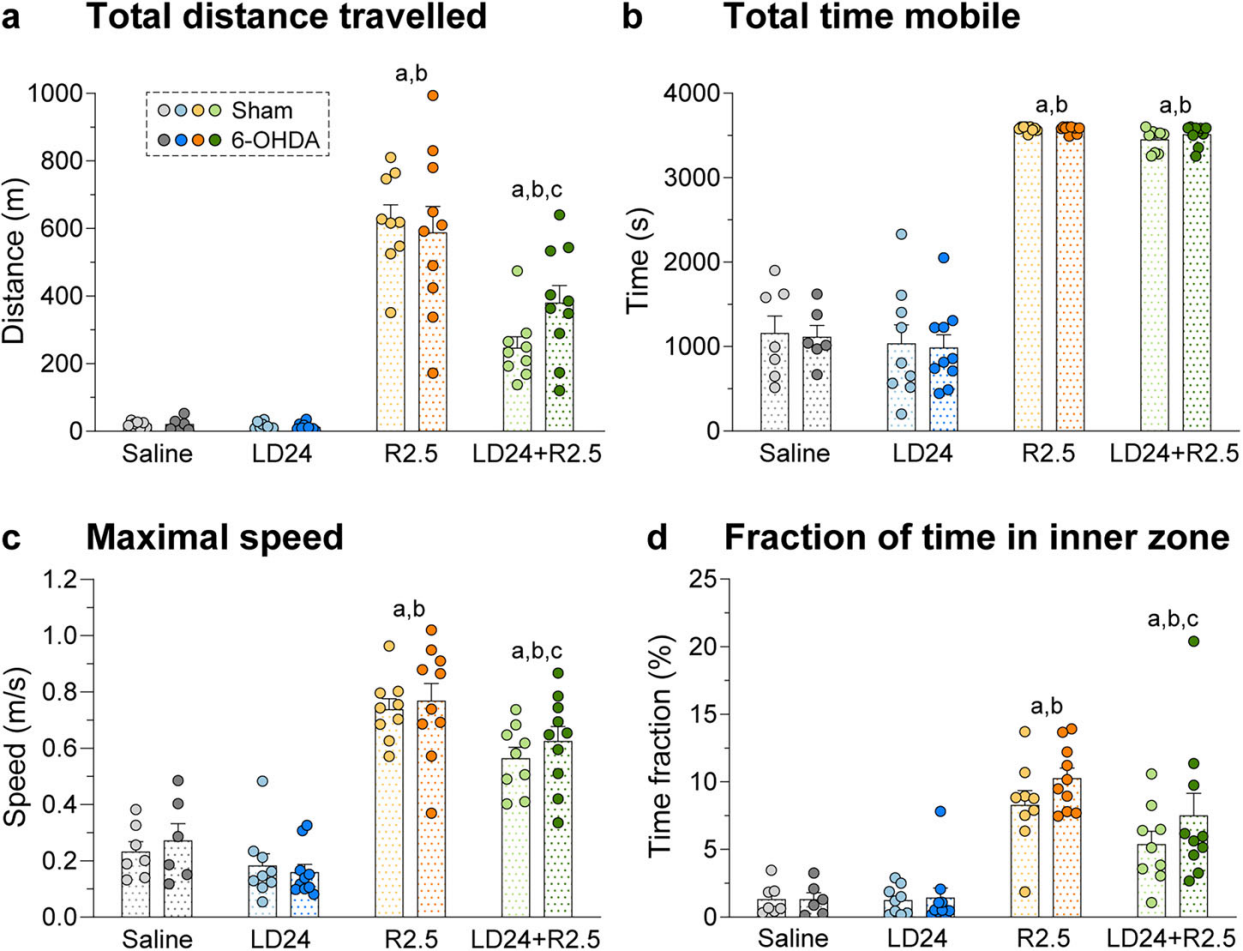
Open field motions. All test parameters were analysed for 60 min after a habituation period of 30 min. Two-factor ANOVAs were followed by Tukey’s post hoc test for the overall treatment effect (*n* = 70). **a** Total distance travelled (m), F(treatment)_3,62_ = 85.8, *p* < 0.001; F(lesion)_1,62_ = 0.6, *p* = 0.445; F(interaction)_3,62_ = 1.6, *p* = 0.200. **b** Total time mobile (s), F(treatment)_3,62_ = 264.9, p < 0.001; F(lesion)_1,62_ = 0.01, *p* = 0.927; F(interaction)_3,62_ = 0.1, *p* = 0.967. **c** Maximal speed (m/s), F(treatment)_3,62_ = 73.0, *p* < 0.001; F(lesion)_1,62_ = 0.6, *p* = 0.426; F(interaction)_3,62_ = 0.3, *p* = 0.805. **d** Fraction of time spent in inner zone (%), F(treatment)_3,62_ = 32.2, *p* < 0.001; F(lesion)_1,62_ = 2.3, *p* = 0.135; F(interaction)_3,62_ = 0.6, *p* = 0.590. Symbols of statistical significance: a = *p* < 0.05 vs Saline; b = *p* < 0.05 vs LD24; c = *p* < 0.05 vs R2.5.

To examine patterns of unconstrained motor activity, animals were video-tracked in an open field arena for 60 min (Fig. 2). Strong effects of the treatment were detected in a two-factor ANOVA when examining the total distance travelled (2a), total time in motion (2b), maximal speed (2c), and the fraction of time spent in the inner zone (2d) (*p* < 0.001 for treatment effect on all four parameters). The same parameters were, however, not affected by the 6-OHDA lesion (*p* > 0.05 for lesion type and treatment x lesion type interaction, further details in Fig. 2 legend).

Treatment with ropinirole induced a large increase in distance travelled in both sham-lesioned and 6-OHDA-lesioned rats (Fig. 2a; >20-fold increase vs saline and LD24, *p* < 0.05). Interestingly, the coadministration of L-DOPA appeared to mitigate this hyperactivity (*p* < 0.05 for R2.5 vs LD24 + R2.5). A similar pattern of treatment effects was found when comparing the groups on maximal movement speed (Fig. 2c; *p* < 0.05 for R2.5 and LD24 + R2.5 vs saline and LD24; *p* < 0.05 for R2.5 vs LD24 + R2.5). Ropinirole also raised the time in motion, and this effect was not modified by L-DOPA cotreatment (Fig. 2b; *p* < 0.05 for R2.5 and LD24 + R2.5 vs saline and LD24).

We then examined the fraction of time spent in the inner zone of the open field, which correlates inversely with anxiety-related behaviours in rodents^33,34^. Interestingly, ropinirole treatment increased the fraction of time in the inner zone approximately four-fold relative to both L-DOPA and saline (Fig. 2d; *p* < 0.05 for R2.5 and LD24 + R2.5 vs saline and LD24). Coadministration of L-DOPA reduced the effect of ropinirole on this parameter (*p* < 0.05 for R2.5 vs LD24 + R2.5).

**Fig. 3:**
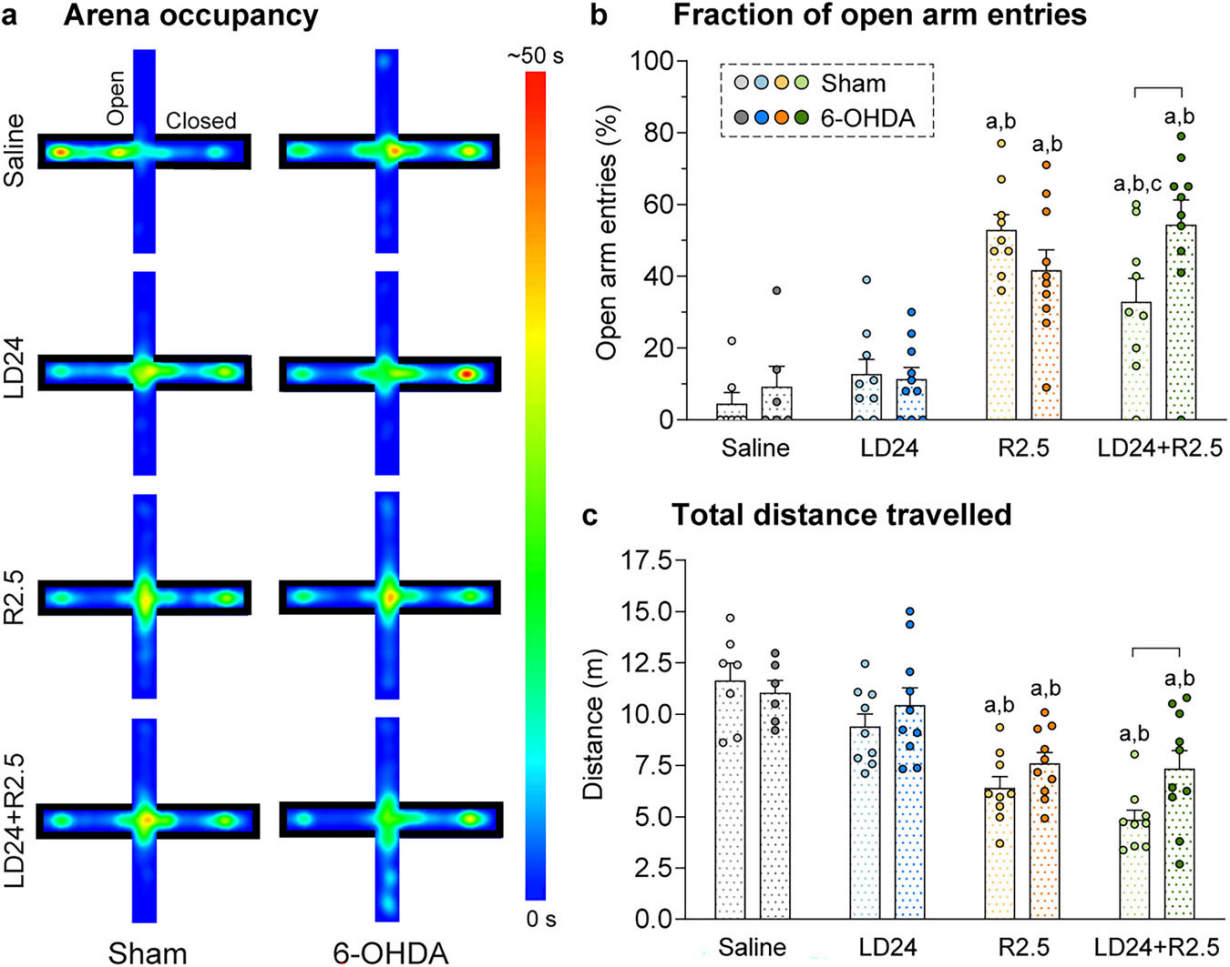
Elevated plus maze (EPM). All test parameters were analysed for the full test period of 5 min. Two-factor ANOVAs were followed by Tukey’s post hoc test for pairwise comparisons within one treatment or lesion type (*n* = 70). **a** Illustrative 2D heat maps of arena occupancy averaged per experimental group in time (s). **b** Fraction of open arm entries (%), F(treatment)_3,62_ = 29.4, p < 0.001; F(lesion)_1,62_ = 0.8, *p* = 0.385; F(interaction)_3,62_ = 3.5, *p* = 0.020. **c** Total distance travelled (m), F(treatment)_3,62_ = 22.3, *p* < 0.001; F(lesion)_1,62_ = 4.2, p = 0.045; F(interaction)_3,62_ = 1.4, *p* = 0.247. Symbols of statistical significance: a = *p* < 0.05 vs Saline within the same lesion type; b = *p* < 0.05 vs LD24 within the same lesion type; c = *p* < 0.05 vs R2.5 within the same lesion type; bracket = *p* < 0.05 for Sham vs 6-OHDA within the same treatment.

Longer time in the open field inner zone suggests reduced levels of anxiety^33,34^. To verify that ropinirole had an anxiolytic-like action, animals were further evaluated in the elevated plus maze (EPM; Fig. 3). The treatment had a strong impact on the fraction of open arm entries in a two-factor ANOVA (*p* < 0.001 for treatment effect), which was also mildly affected by the lesion (*p* < 0.05 for lesion type x treatment interaction, non-significant effect of lesion type alone).

Ropinirole treatment produced a large increase in the fraction of open arm entries in both sham-lesioned and 6-OHDA-lesioned animals, indicating an anxiolytic action (Fig. 3a, b, *p* < 0.05 for R2.5 and LD24 + R2.5 vs saline and LD24). L-DOPA cotreatment tended to slightly reduce this effect only in sham-lesioned animals (*p* < 0.05 for R2.5 vs LD24 + R2.5 in the sham cohort), whereas 6-OHDA-lesioned animals coadministered with ropinirole and L-DOPA did not differ significantly from ropinirole treatment alone (*p* < 0.05 for 6-OHDA- vs sham-lesioned animals under LD24 + R2.5 cotreatment).

During the EPM test, ropinirole did not induce an increase in the total distance travelled as seen in the open field test, but instead reduced the distance travelled compared with both L-DOPA and vehicle treatment, an effect independent of both lesion type and L-DOPA coadministration (Fig. 3c; *p* < 0.05 for R2.5 and LD24 + R2.5 vs saline and LD24). These data rule out that the increase in open arm entries observed under ropinirole treatment might have depended on hyperlocomotion. Additionally, cotreatment with L-DOPA and ropinirole resulted in a larger distance travelled in 6-OHDA- vs sham-lesioned animals (Fig. 3c; *p* < 0.05).

### Ropinirole induced a stereotypic pattern of behavioural activation

**Fig. 4:**
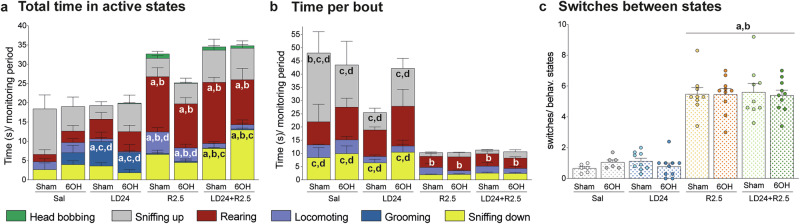
Analysis of active behaviours. Animals were filmed every 20 min for 60 min following treatment with saline in sham- and 6-OHDA-lesioned, *n* = 6 and *n* = 6, LD24 sham- and 6-OHDA-lesioned, *n* = 9 and *n* = 10, R2.5 sham- and 6-OHDA-lesioned, *n* = 9 and *n* = 10, and LD24 + R2.5 sham- and 6-OHDA-lesioned, *n* = 9 and *n* = 10. **a** Total time spent in each state averaged across time points. Two-factor ANOVA Rearing, F(treatment)_3,61_ = 15.3, *p* < 0.001; F(lesion)_1,61_ = 1.1, *p* = 0.302; F(interaction)_3,61_ = 0.7, *p* = 0.530. Sniffing up, F(treatment)_3,61_ = 1.9, *p* = 0.140; F(lesion)_1,61_ = 0.6, *p* = 0.814; F(interaction)_3,61_ = 1.5, *p* = 0.230. Sniffing down, F(treatment)_3,61_ = 12.6, *p* < 0.001; F(lesion)_1,61_ = 0.305, *p* = 0.583; F(interaction)_3,61_ = 2.7, *p* = 0.053. Locomoting, F(treatment)_3,61_ = 15.8, *p* < 0.001; F(lesion)_1,61_ = 1.1, *p* = 0.300; F(interaction)_3,61_ = 1.4, *p* = 0.237. Grooming, F(treatment)_3,61_ = 8.5, *p* < 0.001; F(lesion)_1,61_ = 0.3, *p* = 0.593; F(interaction)_3,61_ = 0.7, *p* = 0.539. Head bobbing, F(treatment)_3,61_ = 2.7, *p* = 0.051; F(lesion)_1,61_ = 1.4, *p* = 0.246; F(interaction)_3,61_ = 1.5, *p* = 0.236. **b** Time spent per bout of states observed in all groups. Two-factor ANOVA Rearing, F(treatment)_3,51_ = 5.1, *p* < 0.001; F(lesion)_1,5_ = 1.2, *p* = 0.271; F(interaction)_3,51_ = 0.5, *p* = 0.673. Sniffing up, F(treatment)_3,55_ = 14.8, *p* < 0.001; F(lesion)_1,55_ = 0.01, *p* = 0.904; F(interaction)_3,55_ = 2.3, *p* = 0.084. Sniffing down, F(treatment)_3,48_ = 9.8, *p* < 0.001; F(lesion)_1,48_ = 1.2, *p* = 0.279; F(interaction)_3,48_ = 0.7, *p* = 0.530. Locomoting, F(treatment)_3,39_ = 1.8, *p* < 0.001; F(lesion)_1,39_ = 0.2, *p* = 0.633; F(interaction)_3,39_ = 0.2, *p* = 0.902. **c** Number of switches divided by number of states present in (**b**) and averaged across the time points. Two-factor ANOVA: F(treatment)_3,61_ = 108.2, *p* < 0.001; F(lesion)_1,61_ = 0.03, *p* = 0.856; F(interaction)_3,61_ = 0.3, *p* = 0.808. Symbols of statistical significance: a = *p* < 0.05 vs Saline; b = *p* < 0.05 vs LD24; c = *p* < 0.05 vs R2.5; d = *p* < 0.05 vs LD24 + R2.5. Abbreviation: 6OH, 6-OHDA-lesioned rats.

DA receptor stimulation in the striatum plays a key role in the initiation and organisation of sequential behaviours^35,36^. To examine the impact of ropinirole and L-DOPA on the animals’ behavioural configuration we used an event recorder software, allowing us to time the start and end of seconds-long behavioural bouts (see “Methods”). The distribution of active time between different behavioural states was strongly affected by the drug treatment, with modest or no impact of the 6-OHDA lesion (Fig. 4a; *p* < 0.001 for the effect of treatment, but *p* > 0.05 for lesion type and lesion x treatment interaction on total time spent rearing, sniffing down, locomoting, and grooming). Indeed, ropinirole-treated animals showed marked increases in both forward locomotion (“locomoting”) and rearing (Fig. 4a; *p* < 0.05 for R2.5 vs LD24 and saline on these behaviours). The addition of LD24 to R2.5 resulted in a reduced representation of forward locomotion (Fig. 4a; *p* < 0.05 for LD24 + R2.5 vs R2.5 6-OHDA and sham) and an increased representation of sniffing behaviour (Fig. 4a; “sniffing down” *p* < 0.05 for LD24 + R2.5 vs R2.5 in the 6-OHDA and sham cohort). Grooming sequences (blue colour) were hardly expressed in animals treated with ropinirole alone or combined with L-DOPA, whereas they accounted for ~25–30% of the total active time in animals treated with L-DOPA (Fig. 4a; *p* < 0.05 for LD24 vs R2.5, LD24 + R2.5 and saline). This result is in agreement with the predominant role of D1 receptors in driving grooming behaviour^36^.

Next, we measured the average duration of active bouts in the four behavioural states that occurred repeatedly in all groups (locomoting, rearing, sniffing down, sniffing up). This analysis, too, revealed a strong overall effect of the treatment, with little or no impact by the lesion (Fig. 4b; *p* < 0.001 for treatment effect on all four behavioural categories, *p* > 0.05 for lesion type and lesion x treatment interaction on locomoting, rearing, and sniffing down). Compared to both L-DOPA and saline groups, animals treated with ropinirole or L-DOPA-ropinirole cotreatment showed a significantly shorter bout duration (usually <5 s/bout) (Fig. 4b; *p* < 0.05 for R2.5 and LD24 + R2.5 vs LD24 and saline for sniffing down and sniffing up states, and *p* < 0.05 for R2.5 and LD24 + R2.5 vs LD24 for rearing).

To determine how frequently animals transitioned between states, we computed the total number of switches between states across the monitoring periods. This analysis revealed that behavioural switching was more frequent in animals treated with ropinirole, whether or not combined with L-DOPA (Fig. 4c; *p* < 0.001 for treatment effect in the two-factor ANOVA; *p* < 0.05 for R2.5 and LD24 + R2.5 vs LD24 and saline in the post hoc comparisons).

In summary, the above results profile the pattern of behavioural activation induced by ropinirole as consisting of a stereotypic repetition of few behaviours (predominantly locomotion, rearing, and sniffing), each expressed in relatively short bouts.

### Ropinirole, but not L-DOPA, induced compulsive checking

**Fig. 5:**
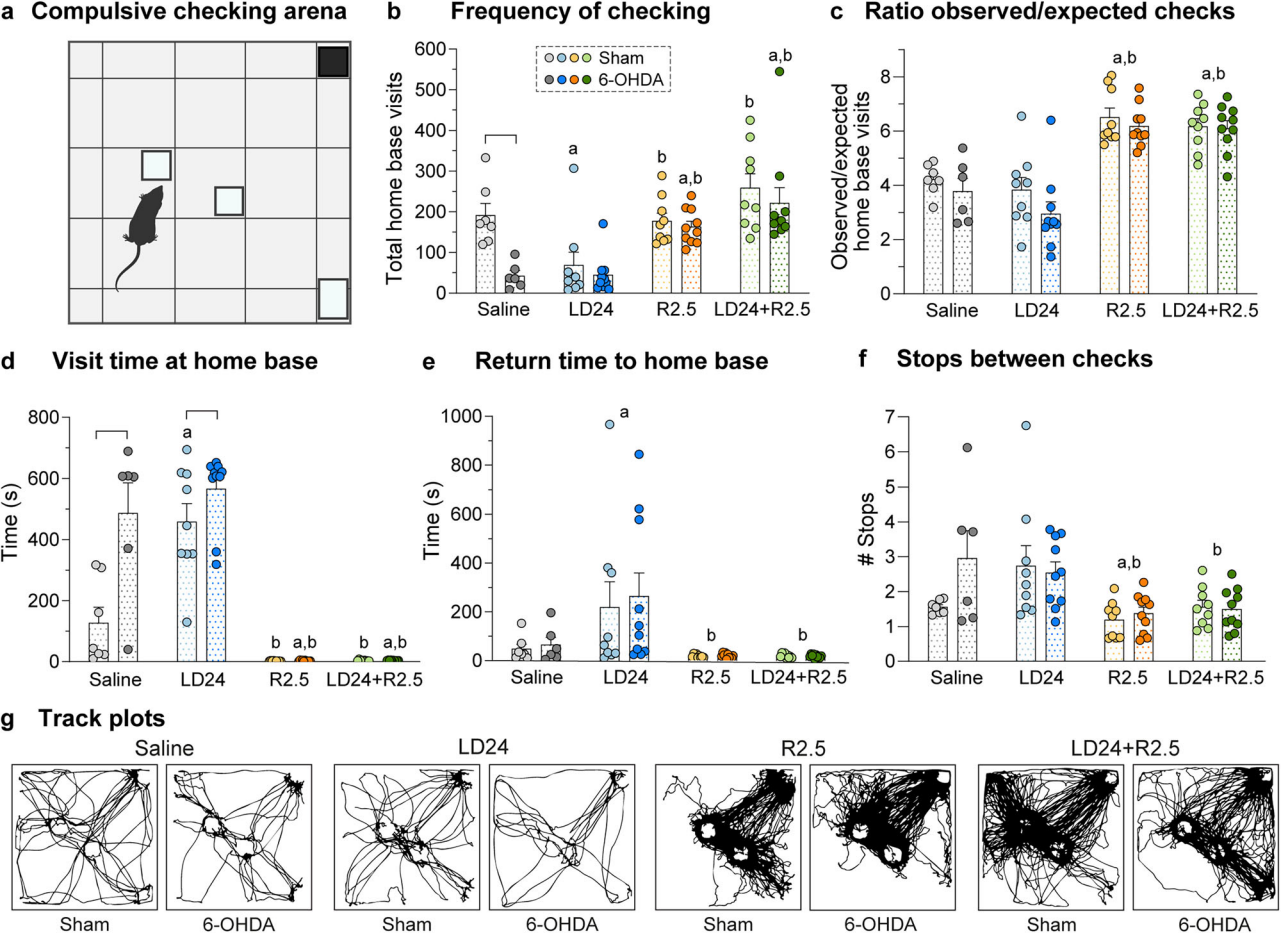
Compulsive checking behaviour. **a** Illustration of the test arena. Squares represent the plexiglass boxes (open on one side) used to elicit checks in specific locales (home bases). **b–f** Parameters used to qualify checking behaviour. Data were analysed with two-factor ANOVAs followed by Tukey’s post hoc test for the overall treatment effect (**c, e, f**) or pairwise comparisons within one treatment or lesion type (**b**, **d**) (*n* = 70). **b** Frequency of checking (total home base visits), F(treatment)_3,62_ = 18.0, *p* < 0.001; F(lesion)_1,62_ = 8.6, *p* = 0.005; F(interaction)_3,62_ = 2.2, *p* = 0.097. **c** Ratio observed/expected home base visits, F(treatment)_3,62_ = 36.9, *p* < 0.001; F(lesion)_1,62_ = 2.9, *p* = 0.096; F(interaction)_3,62_ = 0.5, *p* = 0.681. **d** Mean visit time at home base, F(treatment)_3,62_ = 89.7, *p* < 0.001; F(lesion)_1,62_ = 18.0, *p* < 0.001; F(interaction)_3,62_ = 8.1, *p* < 0.001. **e** Mean return time to home base, F(treatment)_3,62_ = 7.9, *p* < 0.001; F(lesion)_1,62_ = 0.2, *p* = 0.691; F(interaction)_3,62_ = 0.1, *p* = 0.970. **f** Average stops between checks, F(treatment)_3,62_ = 7.3, *p* < 0.001; F(lesion)_1,62_ = 1.8, *p* = 0.183; F(interaction)_3,62_ = 1.9, *p* = 0.141. **g** Track plots of movement in the compulsive checking arena for representative example animals under different treatments. Symbols of statistical significance: a = *p* < 0.05 vs Saline; b = *p* < 0.05 vs LD24; bracket = *p* < 0.05 for Sham vs 6-OHDA within the same treatment.

Compulsive checking behaviour was assessed by characterising the visits to some preferred locales (“home bases”) within a large open field (Fig. 5a, see “Methods” for further details). In the original description of this test, compulsive checking was considered present when the rat returned to the home bases excessively often and rapidly, and visited fewer places along the way^27^. We therefore examined the frequency of checking (5b), the ratio between observed and expected checks, where the frequency of checking was normalised to the total distance travelled (5c), the home base visit time (5d), the return time (5e), and the number of stops between checks (5f).

A significant overall effect of the treatment was detected on all of these measures in a two-factor ANOVA (Fig. 5b–f; *p* < 0.001 for treatment effect on all five parameters; see Fig. 5 legend for details). In contrast, the lesion type had a significant impact only on the frequency of checking and the home base visit time. The frequency of checking tended to be reduced by the 6-OHDA lesion in all treatment arms (Fig. 5b; *p* = 0.005 for lesion type; *p* = 0.097 for lesion x treatment interaction; *p* < 0.05 for 6-OHDA vs sham under saline treatment). The home base visit time was prolonged by the 6-OHDA lesion in animals treated with L-DOPA or vehicle (Fig. 5d; *p* < 0.001 for lesion type; *p* < 0.001 for lesion x treatment interaction; *p* < 0.05 for 6-OHDA vs sham under saline and LD24 treatment).

Both the overall frequency of checking and the ratio of observed vs expected checks were significantly increased by ropinirole, and this effect was not modified by the coadministration of L-DOPA (Fig. 5b, c; *p* < 0.05 for R2.5 and LD24 + R2.5 vs LD24 in both sham and 6-OHDA cohorts; *p* < 0.05 for R2.5 and LD24 + R2.5 vs saline in the 6-OHDA cohort). Additionally, the time spent at home bases was reduced drastically upon ropinirole treatment regardless of L-DOPA cotreatment or lesion type (Fig. 5d; *p* < 0.05 for R2.5 and LD24 + R2.5 vs LD24 in both sham and 6-OHDA cohorts; *p* < 0.05 for R2.5 and LD24 + R2.5 vs saline in the 6-OHDA cohort). Ropinirole treatment also shortened the mean return time to home bases and reduced the average number of stops in between checks (Fig. 5e, f; *p* < 0.05 for R2.5 and LD24 + R2.5 vs LD24; *p* < 0.05 for R2.5 vs saline in the stops between checks parameter).

In summary, rats treated with ropinirole fulfilled all criteria that establish the occurrence of compulsive checking in this test: High frequency of home base visits with short return time and reduced number of stops in other locales. The effects of ropinirole on the measured parameters were largely independent of L-DOPA coadministration and lesion type. Patterns of movement in the arena of representative example animals for each experimental group are shown as track plots in Fig. 5g and as short video clips in Fig. S1.

### Ropinirole, but not L-DOPA, impaired task acquisition in the rIGT

**Fig. 6:**
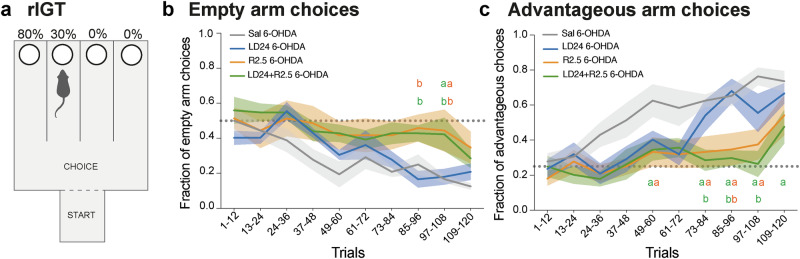
Rat Iowa gambling task. Animals were injected with saline (grey, *n* = 6), LD24 (blue, *n* = 6), R2.5 (orange, *n* = 6), and LD24 + R2.5 (green, *n* = 7) 15 min before initiating each session of 12 trials. **a** Scheme of the rat Iowa Gambling Task (rIGT) arena (see methods for details). **b** Fraction of empty arm choices to the total arm choices per 12 trials (Repeated measurement mixed effect model: F(treatment)_3,21_ = 4.5, *p* = 0.011; F(time)_9,81_ = 11.7, *p* < 0.001; F(interaction)_27,93_ = 1.4, *p* = 0.120). **c** Fraction of advantageous arm choices to the total arm choices per 12 trials (Repeated measurement mixed effect model: F(treatment)_3,27_ = 7.5, *p* < 0.001; F(time)_9,81_ = 12.6, *p* < 0.001; F(interaction)_27,93_ = 1.8, *p* = 0.017). Dotted lines mark the chance level for each analysis. Symbols of statistical significance: a = *p* < 0.05 vs Saline; b = *p* < 0.05 vs LD24; orange and green letters represent R2.5 and LD24 + R2.5 treatments, respectively.

The Iowa gambling task is commonly used to assess impulsive decision-making^28,37^, and PD patients on dopaminergic medications show significant impairments in this task^38^. To model the IGT in rats, we used a task where animals gradually learn to choose the option delivering maximum long-term reward with minimal losses over repeated trial blocks^39^. The test apparatus consists of a start box, a choice area, and four goal arms, two of which are baited with sugar pellets and quinine-saturated pellets with different probabilities, while two are left empty (Fig. 6a; further details in “Methods”). The rIGT test protocol was initiated in the fourth week of drug treatment (see timeline in Fig. 1a) and was only applied to the 6-OHDA-lesioned cohort due to logistics constraints.

We first assessed if the animals could learn to discriminate between empty and baited arms and detected an overall difference between treatments (Fig. 6b, *p* < 0.05 for treatment effect; *p* < 0.001 for time effect; *p* = 0.120 for time x treatment interaction). While animals treated with saline or L-DOPA exhibited a steady reduction in empty arm choices from the early trial blocks (1–60), those treated with ropinirole or the combination of L-DOPA and ropinirole continued to perform at close to chance levels during the majority of test trials (Fig. 6b; *p* < 0.05 for R2.5 and LD24 + R2.5 vs. LD24 in trial blocks 85–96, and *p* < 0.05 for R2.5 and LD24 + R2.5 vs. LD24 and saline in trial blocks 97–108). However, in the last 12 trials (109–120), no significant group difference was detected.

Next, we investigated the animals’ ability to discriminate the arm associated with largest long-term reward (80% pellet wins) from the other three arms (Fig. 6c). Starting from the third trial block (24–36), animals treated with saline showed an increasing preference for the most advantageous arm, exhibiting a steady improvement above chance level (depicted as a dotted grey line in Fig. 6c). L-DOPA-treated rats showed a somewhat delayed learning curve, although they did not differ significantly from saline controls in any trial block. In contrast, animals treated with ropinirole and the combination of L-DOPA and ropinirole performed close to chance level from the beginning of the test through the second-to-last trial block (trials 97–108) (Fig. 6c; p < 0.05 for R2.5 and LD24 + R2.5 vs saline on trials 49–60, 73–84, 85–96, 97–108, *p* < 0.05 for LD24 + R2.5 vs LD24 on trials 73–84, 85–96, 97–108, and *p* < 0.05 for R2.5 vs L-DOPA on trials 85–96). In the last trial block (109–120), ropinirole-treated animals no longer differed significantly from the saline- and L-DOPA-treated groups, despite their fraction of advantageous choices not quite reaching the same high values (Fig. 6c). Animals receiving the combined treatment of ropinirole and L-DOPA, however, maintained a significant impairment relative to saline-treated controls (Fig. 6c, *p* < 0.05 for LD24 + R2.5 vs saline on trials 109–120).

In summary, whether given alone or combined with L-DOPA, ropinirole treatment affected the animals’ ability to establish an advantageous choice strategy negatively in this task. In contrast, treatment with L-DOPA alone did not cause a significant impairment.

### Striatal neuroactivity patterns as defined using pS6 as a marker

**Fig. 7:**
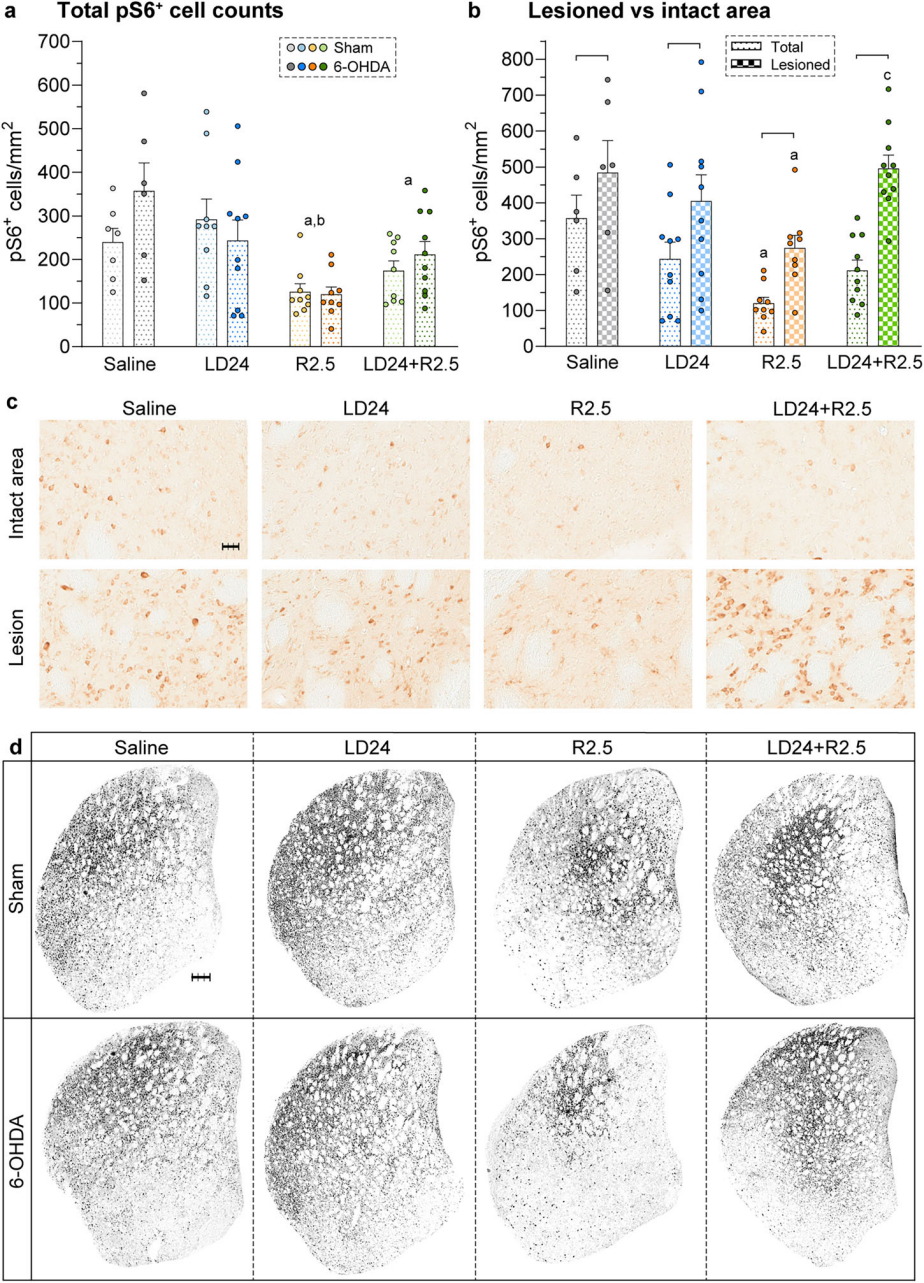
Striatal expression of pS6, a marker of neuronal activation. **a** pS6-immunopositive (pS6^+^) cell counts in the whole striatum under different treatments (*n* = 69). Two-factor ANOVA was followed by Tukey’s post hoc test for the overall treatment effect. F(treatment)_3,61_ = 8.9, *p* < 0.001; F(lesion)_1,61_ = 0.9, *p* = 0.339; F(interaction)_3,61_ = 1.7, *p* = 0.183. **b** pS6^+^ cell counts in the whole striatum and the denervated area in 6-OHDA-lesioned animals under different treatments (*n* = 35). Two-factor repeated measurement ANOVA was followed by Tukey’s post hoc test for pairwise comparisons within one treatment or lesion type. F(treatment)_3,31_ = 3.3, *p* = 0.033; F(area)_1,31_ = 210.0, *p* < 0.001; F(animal)_31,31_ = 15.9, *p* < 0.001; F(area x treatment)_3,31_ = 8.4, *p* < 0.001. **c** Representative immunohistochemical stainings of pS6^+^ cells in the striatum developed using 3,3’^-^diaminobenzidine (DAB). Shown are high magnification images of the intact and the lesioned area of one example animal per treatment. Scale bar: 30 μm. **d** Representative immunohistochemical stainings of pS6^+^ cell distributions in the striatum developed using DAB from one example animal per experimental group. Scale bar: 300 μm. Symbols of statistical significance: a = *p* < 0.05 vs Saline; b = *p* < 0.05 vs LD24; c = *p* < 0.05 vs R2.5; bracket = *p* < 0.05 for Total vs Lesioned area within the same treatment.

Serine-phosphorylated pS6 has been used as a molecular marker of neuronal activation in a wide variety of brain regions, neuronal types, and experimental conditions^30,31^. We therefore chose this marker to explore activity changes affecting striatal neurons after the different treatments. In the total striatal area, counts of phosphorylated pS6-immunopositive cells (pS6^+^ cells) were markedly affected by the pharmacological treatment (*p* < 0.001), but not by the 6-OHDA lesion (*p* > 0.05 for lesion type and lesion x treatment interaction) in a two-factor ANOVA (Fig. 7a).

In particular, ropinirole treatment decreased the count of pS6^+^ neurons relative to both saline and L-DOPA treatment (Fig. 7a, c, intact area; ~60% and 55% reduction after R2.5 vs saline and LD24 treatment, respectively; *p* < 0.05 in both 6-OHDA and sham cohorts). Cotreatment with L-DOPA tended to mitigate this reduction as animals under cotreatment had larger average cell counts and did not differ significantly from the L-DOPA group (but *p* < 0.05 for LD24 + R2.5 vs saline).

We next compared DA-denervated and DA-intact regions of 6-OHDA-lesioned rats and found a significant increase in pS6^+^ cell counts in TH-depleted areas in each treatment group (Fig. 7b, c; *p* < 0.05 for lesioned vs total area in all groups). The relative increase in cell counts in denervated regions was most prominent under the cotreatment with L-DOPA and ropinirole (approximately +140%), followed by the ropinirole treatment alone ( +128%). Yet, ropinirole-treated animals had low pS6^+^ cell counts also within the denervated area compared to the other groups (Fig. 7b; *p* < 0.05 for R2.5 vs saline in the lesioned area).

Reasoning that localised effects of lesion and treatment would not be captured by total pS6^+^ cell counts in the striatum, we created 2D histograms of pS6^+^ cell distributions in each experimental group (Fig. S2). Pixelwise comparisons of pS6^+^ cell counts across groups revealed significant differences dependent on the treatment, with an additional impact of the lesion in TH-depleted areas (see statistical maps in Fig. S2). Compared to saline and L-DOPA, ropinirole alone or combined with L-DOPA resulted in an overall reduced activation of the dorsolateral striatum and a shift of maximal pS6^+^ cell counts towards central and medial coordinates. The ropinirole-related pattern was evident in both sham-lesioned and 6-OHDA-lesioned animals, which, however, displayed localised areas with higher pS6^+^ cell density within the TH-depleted region (see actual distribution maps in Fig. 7d and the statistical maps in Fig. S2).

To better examine coactivation patterns associated with different conditions, we performed principal component analysis (PCA) on the 2D histograms from all experimental conditions combined. The first three principal components (PCs) clearly showed a differential expression depending on condition (Fig. S3), explaining about 33% of the overall variance in the material, and were therefore investigated further to determine how much they contributed to the activation pattern of each experimental group.

**Fig. 8:**
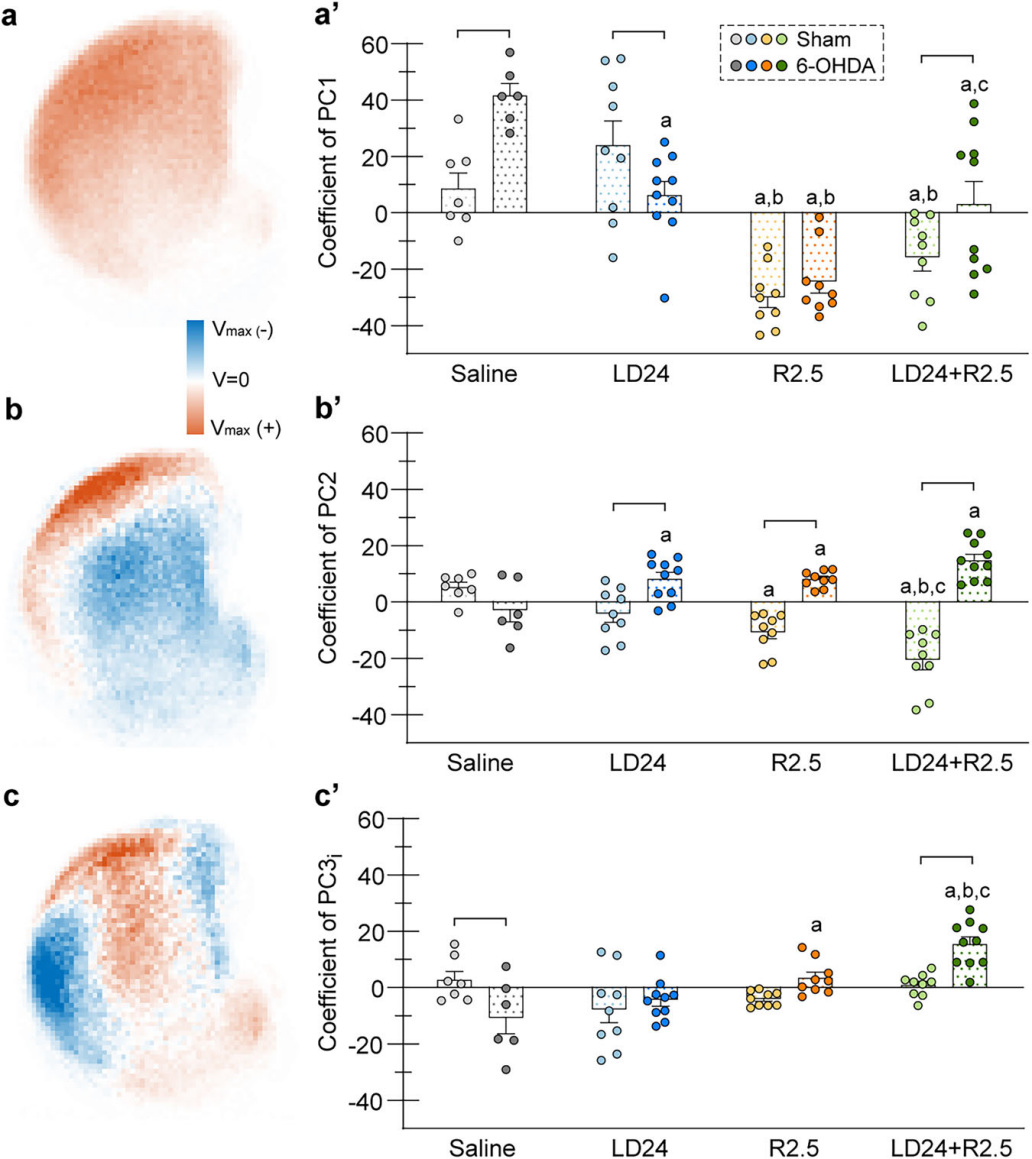
Patterns of striatal neuroactivity studied by principal component analysis. **a–c** Main principal components (PCs) identified in a principal component analysis of 2D histograms of pS6^+^ cell distributions in the striatum. Colour scale shows local variance, V_max_ = maximal variance. Covariance is present in pixels with the same variance sign (positive: red, or negative: blue) and antivariance is present in pixels with opposite variance signs (red vs blue). **a** PC1. **b** PC2. **c** Inverted PC3. **a’–c’** Coefficients of main PCs as indices of the expression level of each covariance pattern for pS6^+^ cell distributions in different experimental groups. Two-factor ANOVAs were followed by Tukey’s post hoc test for pairwise comparisons within one treatment or lesion type; n(independent animals)=69, n(sections)=411. **a’** Coefficient of PC1. F(treatment)_3,61_ = 28.3, *p* < 0.001; F(lesion)_1,61_ = 5.3, *p* = 0.024; F(interaction)_3,61_ = 6.1, *p* = 0.001. **b’** Coefficient of PC2. F(treatment)_3,61_ = 1.6, *p* = 0.191; F(lesion)_1,61_ = 62.3, *p* < 0.001; F(interaction)_3,61_ = 21.7, *p* < 0.001. **c’** Coefficient of inverted PC3. F(treatment)_3,61_ = 10.0, *p* < 0.001; F(lesion)_1,61_ = 2.1, *p* = 0.155; F(interaction)_3,61_ = 7.3, *p* < 0.001. Symbols of statistical significance: a = *p* < 0.05 vs Saline within the same lesion type; b = *p* < 0.05 vs LD24 within the same lesion type; c = *p* < 0.05 vs R2.5 within the same lesion type; bracket = *p* < 0.05 for Sham vs 6-OHDA within the same treatment.

The covariance pattern captured by the first component (PC1) corresponded to highest pS6^+^ cell counts in the dorsolateral quadrant and flanking the external capsule, gradually declining along the dorsoventral and mediolateral axis (Fig. 8a). Positive expression of PC1 was found in saline- and L-DOPA-treated groups, whereas strongly negative expression was seen in animals treated with ropinirole (Figs. 8a’ and S3a; *p* < 0.05 for treatment, lesion type, and treatment x lesion interaction in two-factor ANOVA; *p* < 0.05 for R2.5 vs saline and LD24). Expression of PC1 was highly variable among animals cotreated with L-DOPA and ropinirole, particularly in the 6-OHDA cohort (*p* < 0.05 LD24 + R2.5 vs R2.5).

The second component (PC2) reflected high pS6^+^ cell counts in the TH-depleted region in anticorrelation to the expression levels across the rest of the striatum (Fig. 8b–b’ and Fig. S3b; *p* > 0.05 for treatment effect and *p* < 0.05 for lesion type and treatment x lesion interaction in two-factor ANOVA). Accordingly, PC2 expression was significantly larger in 6-OHDA- compared with sham-lesioned animals in all treatment arms except for saline (Fig. 8b’; *p* < 0.05 for 6-OHDA vs sham in LD24, R2.5, and LD24 + R2.5). 6-OHDA-lesioned animals treated with L-DOPA and ropinirole, alone or combined, showed a positive expression of PC2 in contrast to the negative expression elicited by the same treatments in sham-lesioned animals. The largest disparity in PC2 expression between sham and 6-OHDA lesions occurred in animals under L-DOPA and ropinirole cotreatment (*p* < 0.05 for LD24 + R2.5 sham vs saline, LD24 and R2.5).

The third component (PC3) captured a localised area with high pS6^+^ cell counts in the TH-depleted region correlated with high activity in central and ventromedial coordinates (Fig. 8c-c’ and Fig. S3c; *p* < 0.001 for treatment and lesion x treatment interaction, *p* > 0.05 for lesion type). A strong positive expression of PC3 was seen only in 6-OHDA-lesioned animals treated with L-DOPA and ropinirole together (Fig. 8c’; *p* < 0.05 for LD24 + R2.5 6-OHDA vs sham and all other treatments).

In summary, this spatial analysis of pS6^+^ cell distribution demonstrated clearcut differences between L-DOPA and ropinirole treatment with regard to patterns of striatal neuroactivity, and moreover revealed a distinct activation pattern in the combined treatment arm, suggesting that adding L-DOPA to ropinirole might modify some of its neurobiological effects.

## Discussion

ICBs are a common and often under-recognised problem in PD^10^, entailing disability, distress, and high caregiver burden^40,41^. Pathophysiological and therapeutic research on the subject lags far behind the advancements made in the study of therapy-related motor complications^16,42^. The number of experimental studies on ICBs in PD is relatively limited, which is partly due to the need for lengthy and sophisticated test protocols to assess altered decision-making and response inhibition in animals^16^. The present study was undertaken to compare ICB features induced by a D2/3 agonist and L-DOPA, alone or combined, with a selection of behavioural methods not previously used in the ICB literature.

We chose to conduct the study in animals with mild dopaminergic lesions for several reasons. First, D2/3 agonists are commonly used as front-line therapy for early-stage PD^43,44^, during which the loss of nigrostriatal dopaminergic innervation is still limited^45–47^. Accordingly, in the experimental literature on Parkinson’s ICBs, ropinirole and pramipexole have been typically given to animals with an intact or only partially damaged dopaminergic system^16^.

Second, the chosen 6-OHDA lesion represents the most common PD model so far used to mimic therapy-induced ICBs in the rat^20,22–26^. The limited extent of striatal DA denervation produced by this lesion is suitable to prevent the occurrence of dyskinesia upon treatment with L-DOPA^16^. Based on previous studies^48–50^, we had indeed estimated that a 70% loss of DA innervation in the lateral striatum would cause most animals to develop dyskinetic behaviours upon treatment with L-DOPA at doses ≥12 mg/kg/day. However, we acknowledge that there are alternative models of partial nigrostriatal degeneration suitable to study ICB in rats, in particular those obtained using viral overexpression of alpha-synuclein in the substantia nigra pars compacta (reported to cause 50–60% dopaminergic cell loss)^19,51–53^. When evaluated at the striatal level, this alpha-synuclein model was found to produce an average 60–68% depletion of DA transporter immunoreactivity across medial and lateral striatum^19^.

Upon immunohistochemical analysis, our lesion was found to produce conspicuous, though localised, damage to the DA fibre network in the dorsolateral striatum at mid-rostrocaudal levels. This region corresponds to the main body of the rat caudate-putamen, strongly innervated by motor and somatosensory cortices^54^. The overall loss of striatal TH immunoreactivity in the caudate-putamen was approximately 30%, being lower than the one reported by previous studies using nominally the same lesion model and where quantitative TH analysis was provided^20^. In line with the localised pattern of striatal dopaminergic denervation, the depletion of TH-positive neurons in the substantia pars compacta was quite modest.

A loss of dopaminergic fibres confined to the dorsolateral striatal quadrant may not accurately mimic the more widespread striatal DA denervation detected in early PD, where a 30-50% overall loss of dopaminergic markers has been detected in the putamen using different molecular imaging methods^55–57^. Despite this localised denervation pattern, the 6-OHDA-lesioned animals showed clearcut forelimb akinesia, which was improved by both L-DOPA and ropinirole treatment. Thus, the extent of DA denervation was sufficient to induce a hypokinetic motor impairment responsive to dopaminergic treatment. The percentage deficit in forelimb use was similar to the one reported by previous studies using this type of lesion^22,26^ and more severe than the one found using more generalised lesion models^51,52^. We therefore hypothesise that our lesion strongly affected the striatal region innervated by cortical motor areas controlling the forelimb^58^, although the denervated area also included additional topographic subdivisions (in particular, trunk and hindlimb regions, see ref. ^59^). At the cellular level, the functional impact of the lesion was documented by an increased number of pS6-immunopositive cells in TH-depleted areas, possibly analogous to the local hypermetabolism found in DA-denervated putaminal regions in PD^60^.

In the tests examining drug-induced behavioural patterns, DA-depleted animals achieved results similar to their DA-intact control group, and differences were detected only in isolated comparisons, such as a reduced expression of forward locomotion and augmented expression of upward sniffing under combined L-DOPA and ropinirole treatment compared with ropinirole treatment alone. These findings are in line with previous investigations utilising the same lesion model, which did not find a difference between 6-OHDA-lesioned and intact animals in the response to ropinirole or pramipexole^23,24,61^. In contrast, other studies have shown a stronger induction of ICB features by DA agonists under DA-depleted conditions^19–21^. One of these studies^20^ used the same 6-OHDA lesion procedure as applied here. The other two studies^19,21^ used animals with extensive nigrostriatal lesions. In the present work, we opted not to use animals with severe nigrostriatal lesions because most of our tests relied on measuring the animals’ spontaneous and self-initiated behaviours in open spaces, in the absence of incentive or aversive stimuli. These behaviours would have been affected by severe hypokinetic deficits or drug-induced dyskinesias interrupting normal motor actions^62^.

The doses of ropinirole and L-DOPA used for chronic treatment in this study had been carefully selected based on both pilot observations and literature review. In our initial dose-response study, the 24 mg/kg L-DOPA dose was found to be well-tolerated and did not induce any dyskinetic or dystonic features. This dose was moreover concordant with those used to induce behavioural effects in rats with partial dopaminergic denervation^63,64^ (which require higher L-DOPA dosages than animals with complete lesions^49^).

In the experimental neuropsychiatric literature, bolus doses of ropinirole in rats have commonly ranged between 0.5 and 10 mg/kg^65–74^, with some studies using even 20–30 mg/kg ropinirole without reporting any adverse effects^66,67,72^. Although these doses are higher than those used clinically, it should be considered that ropinirole has much faster elimination kinetics in rodents compared with humans^75,76^, and that upward adjustments are needed when translating human doses to rodents^77^. Some publications examining ropinirole’s ICB-inducing effects in rats have used a daily dose of 5 mg/kg, though administered continuously via osmotic minipumps over the behavioural training weeks^24,61,76,78^. This administration method would not have been suitable for the present work, where all behavioural recordings were timed to start 15 min after drug administration.

After evaluating the dose range 0.5–5.0 mg/kg in a pilot experiment, we selected 2.5 mg/kg ropinirole for the chronic study because it induced compulsive checking behaviour without causing any motor adverse effects. Moreover, 2.5 mg/kg ropinirole was well aligned with the pramipexole dose (3 mg/kg s.c.) reported to increase impulsivity in rats subjected to the same 6-OHDA lesion used here^20^. Ropinirole and pramipexole have marked pharmacological and clinical similarities^79,80^. They both have high selectivity for D2 and D3 receptors, where ropinirole has a comparable activity on these two receptor types while pramipexole has an 8–10 times stronger affinity for D3 over D2^81^. Clinical studies have shown that pramipexole has a longer plasma elimination half-life than ropinirole (8–12 vs 3–6 h, respectively^1,80^) and it is used at lower daily dosages^80–82^.

The results from the open field test and behavioural state analysis are concordant in showing a strong stimulation of locomotion by ropinirole in both 6-OHDA-lesioned and intact animals. Previous studies in rats treated with ropinirole^73^ or other high-affinity D2-class receptor agonists^83–85^ have reported either a reduction or no change in locomotion using drug doses below 0.5–1.0 mg/kg, although locomotor activation was seen with higher doses^73,83^. It is well known that D2/3 agonists have a biphasic effect on motor activity, as low doses mainly induce motor inhibition, which is replaced by motor activation as dosages increase^83,84^. This biphasic effect reflects two independent actions of D2/3 agonists, that is, the activation of presynaptic autoreceptors on DA neurons (leading to motor inhibition) and the stimulation of postsynaptic receptors on dopaminoceptive neurons (leading to motor activation). Our results show that the 2.5 mg/kg ropinirole dose was clearly sufficient for the postsynaptic action to prevail over the presynaptic one.

Interestingly, the open field test data show that L-DOPA cotreatment mitigated the hyperlocomotion induced by ropinirole. Indeed, both the distance travelled and the maximal speed were lower in the combined treatment group compared with the one treated with ropinirole only. This effect of L-DOPA is likely to reflect a stimulation of D1 receptors, which has been shown to alter the pattern of D2 agonist-induced locomotion in rats by increasing the duration of pauses between bouts of activity^86^.

Interestingly, the induction of hyperlocomotion by ropinirole was accompanied by an anxiolytic-like effect, as the time spent in the centre of the open field arena was increased. An anxiolytic effect was moreover confirmed by the large increase in open arm entries induced by ropinirole in the EPM test. These results are in keeping with previous studies showing an anxiolytic action by D2/3 agonists in rodent models of bilateral partial DA denervation^50,87–90^ as well as intact rats^73^. Supporting the clinical relevance of these findings, ropinirole has been reported to ameliorate depressive and anxious symptoms in PD patients with akinetic-rigid phenotypes and dyskinesias^91,92^.

The strong stimulation of locomotion induced by ropinirole is unlikely to purely reflect a motor effect. In some preclinical models, hyperlocomotion is used as an index of neuropsychiatric dysfunction (see e.g., refs. ^93,94^.). In our study, ropinirole-induced hyperlocomotion was part of a stereotypic behavioural pattern including repetitive rearing and sniffing, which is indicative of a neuropsychiatric involvement extending beyond the motor circuits. Accordingly, ropinirole has been shown to alter neuronal activity in the same corticostriatal limbic networks that regulate motivational states and substance abuse^73^. It is also relevant to mention that drugs of abuse, like cocaine or methamphetamine, increase locomotion in rodents^95–101^.

Our behavioural classification analysis showed that ropinirole, given alone or combined with L-DOPA, induced a restricted number of behaviours that were repeated as short bouts between and within the hyperlocomotive periods. A frequent repetition of specific behaviours with little variation is indicative of compulsivity^102^ and may have analogies with some aspects of ICBs in PD, such as punding^16,41^.

Szechtman and colleagues were the first to report that chronic treatment of intact rats with the D2/3 agonist quinpirole induces excessive repetitive checking of particular locales (“home bases”) within a large open field arena^27^. Thereafter, several studies have used this test paradigm to study rodent models of obsessive-compulsive disorder^103–106^. In the present study, the Szechtman test is applied for the first time to mimic Parkinson’s ICBs. Our results demonstrate that compulsive checking is induced by ropinirole, but not L-DOPA, both in DA-intact and partially DA-depleted animals. Specifically, animals treated with ropinirole revisited their home bases excessively often and stopped at fewer places between the visits. This spatiotemporal structure fulfils the definition of compulsive features, probed accordingly with the Yale-Brown Obsessive Compulsive Scale in patients^107^, indicating a subject’s preoccupation with the item of interest and its reluctance to leave it^27^. Importantly, the cognitive and neural mechanisms underlying compulsive behaviours have been suggested to overlap across disorders^108,109^. Thus, although the test was originally developed to study checking behaviour in obsessive-compulsive disorders, the measured construct might share similarities with compulsive features of common ICBs experienced by PD patients.

Maladaptive behaviours characterised by repeated and excessive engagement in specific activities, such as problem gambling, compulsive sexuality, excessive hobbyism, and punding, are part of the ICB spectrum observed in PD patients^16^. Like all ICBs, these features are more prevalent in PD patients treated with DA agonists compared to L-DOPA^10,14,110,111^. However, the association of compulsive behaviours with combined dopaminergic pharmacotherapies has been less clear and remains a matter of debate. While some studies reported an increased risk of compulsive behaviours in PD patients receiving combination therapies that include L-DOPA and a DA agonist^14,112^, several other studies found a similar or even lower risk under polytherapy including L-DOPA compared with DA agonist monotherapy^10,110,113^. On this matter, our findings support a major risk for repetitive, non-goal-oriented behaviours when using D2/3 agonists, and a much lower risk when using L-DOPA.

To study impulsive features, we have here utilised the non-operant maze-based version of the rIGT. This test assesses decision-making in a context of conflict between immediate and long-term payoffs^39^. Establishing a successful strategy in the rIGT engages several affective and cognitive functions that are also involved in human decision-making^28,37^. These include attention, working memory, motivation for reward, and sensitivity to risk and punishment^114^.

Due to logistics constraints, the rIGT protocol was carried out only in the 6-OHDA lesion cohort. Notwithstanding this limitation, comparisons with previous studies on intact rats indicate that the saline-treated lesioned animals learned the task as expected, showing a progressively lower fraction of empty arm choices accompanied by a steady increase in the fraction of advantageous choices along the test protocol^39,115,116^.

Our results show that ropinirole, alone or coadministered with L-DOPA, markedly delayed the animals’ ability to learn the task. Indeed, treatment with ropinirole alone or combined with L-DOPA impaired the rats’ ability to choose the advantageous arm through the second-to-last trial block, and animals in the combined treatment group maintained a significant difference from saline controls through the end of the test protocol. In contrast, animals treated with L-DOPA alone did not differ significantly from the saline control group in any trial block. These results are in agreement with previous rodent studies showing a deleterious effect of D2/3 agonist treatment in tasks of decision-making under uncertainty of outcomes^22,23,117,118^, also including operant versions of the IGT^119^.

Treatment-induced differences in choice strategies were more evident during the second half of the test protocol (trials 73–108), when the animals were expected to transition from random exploration to establishing a preference for the maze arm yielding a larger long-term reward^39,120,121^. It has been proposed that these later stages of the task depend more strongly on the cognitive corticostriatal loops (encompassing prelimbic cortex and dorsomedial striatum) compared with the emotional ones (orbitofrontal cortex, infralimbic cortex, and nucleus accumbens)^28^. To perform successfully in the late stages of the task, animals need to strengthen the long-term advantageous option and weaken the disadvantageous ones^28^. Dopaminergic medications have been shown to impair the patients’ ability to learn from negative decision outcomes^122^, a task heavily dependent on the indirect basal ganglia pathway originating from D2 receptor-positive striatal neurons^35^. It is therefore not surprising that a treatment achieving strong stimulation of D2 receptors, thus inhibiting indirect pathway neurons, impairs the cognitive components of the rIGT and the underlying decision-making process. That ropinirole induced an impaired sensitivity to risk is also suggested by the activity patterns recorded in the open field and EPM tests, where animals receiving this treatment spent a larger proportion of time in zones that rodents normally perceive as dangerous.

Phosphorylated pS6 is a well-established marker of neuronal activity induced by dopaminergic stimulation^31^. Unlike c-Fos, the induction of pS6 is not subject to desensitisation upon repeated stimulation, thus being especially suitable for monitoring neuronal changes during chronic drug treatments^30,123^. The mapping of pS6-positive cells in the striatum showed an overall reduction under ropinirole treatment, as expected from a DA agonist that activates an inhibitory signalling pathway^124^. The reduction effect was mitigated by the coadministration of L-DOPA, in keeping with L-DOPA treatment also stimulating D1 receptors.

To more accurately investigate patterns of striatal neuroactivity, we carried out a PCA on the distribution patterns of cellular pS6 expression. The three most influential principal components provided a “fingerprint” of how different treatments activated the striatum. While animals treated with L-DOPA or saline showed predominant activity in the dorsolateral striatum, ropinirole alone or combined with L-DOPA shifted the activity to central and medial areas. A well-accepted, functional compartmentalisation of the striatum holds that goal-directed behaviours are under the control of the dorsomedial caudate-putamen and the nucleus accumbens, while the acquisition and implementation of habitual actions are dorsolateral-dependent^125^. The observed shift of neuronal activation towards medial areas induced by ropinirole relative to L-DOPA suggests preferential recruitment of striatal pathways involved in behavioural choice rather than mere motor action. These observations are relevant to understanding ropinirole’s propensity to affect decisional ability and behavioural flexibility.

Interestingly, the study of pS6 expression demonstrated a clear effect of the adjunct L-DOPA treatment on the striatal activation pattern induced by ropinirole, particularly in the 6-OHDA lesion cohort. This observation shows the utility of combining behaviour with cellular mapping studies to gain insight into neuroplasticity patterns induced by different PD treatments.

In conclusion, using a novel combination of behavioural analyses in an animal model of early parkinsonism, the present study shows that enhanced compulsivity, impulsive decision-making, and stereotypic movement patterns are equally induced by a D2/3 agonist when administered alone or combined with L-DOPA. These results would suggest that adding L-DOPA to a D2/3 agonist does not entail a higher risk for ICBs, at least in the setting of early dopaminergic degeneration. Our suggestion is in keeping with the results of longitudinal cohort studies investigating the relationship between DA replacement therapy and impulse control disorders (ICDs) in PD, which did not find a significant interaction between DA agonists and L-DOPA, nor any strong association between ICDs and L-DOPA use in general^10,113^. Similar conclusions were presented in a cross-sectional population-based study conducted on 125 PD patients in Norway^110^. By contrast, a large cross-sectional study involving 3090 people with PD in Northern America (The DOMINION study^14,126^) reported an increased ICD risk in patients receiving combined treatment with DA agonists and L-DOPA, and a correlation between heightened ICD risk and the use of higher L-DOPA doses^14^. Moreover, a prospective 2-year study on 1095 people with PD in Italy reported a larger ICD prevalence comparing patients on DA agonist-L-DOPA cotreatment with those receiving DA agonists or L-DOPA alone (ICARUS study^127^). As the Authors themselves explain, patients were classified according to the PD therapy that was ongoing in the 4 weeks prior to a given visit (3 visits over 2 years) and some of the recorded ICDs may have been reflective of a previous therapy (i.e. received for ≥4 weeks prior to the visit)^127^. This comment is important because longitudinal studies have shown that the prevalence of ICDs declines very slowly after discontinuation of DA agonist treatment^10^. In summary, the medication adjustments frequently experienced by ICD patients (typically, DA agonist tapering and introduction of L-DOPA) are difficult to control for using cross-sectional patient assessments, which are vulnerable to reverse causation^10,14,113,126,127^. Reverse causation would occur if the tapering of DA agonist treatment due to ICDs leads to a secondary increase in L-DOPA dosage, prompting the conclusion that excess L-DOPA dosage is the cause of ICDs^10,113^. Having said that, it should also be emphasised that L-DOPA itself can induce behaviours with compulsive features in vulnerable individuals, such as DA dysregulation syndrome and punding (commented upon in refs. ^16,128^). These have been reported to concur with typical ICDs in several studies^129,130^.

From a neurobiological perspective, combining a D2/3 agonist with L-DOPA appears like an advantageous strategy to achieve antiparkinsonian benefits with a lower risk of both ICB and motor complications. The PCA of pS6 cell distributions indeed suggests that combining the two treatment principles produces a more balanced pattern of striatal activation, engaging both dorsolateral (motor) and medial-ventral regions (cognitive-limbic). In addition, the mitigation of ropinirole-induced hyperlocomotion seen upon L-DOPA cotreatment suggests that combining the two drugs results in a more balanced pattern of D1-D2 receptor class stimulation. Supporting the benefits of combination therapies, recent studies focused on motor function have shown that combined treatment with ropinirole and low-dose L-DOPA achieves the same level of motor improvement as a full-dose L-DOPA regimen without inducing dyskinesia and the associated markers of maladaptive striatal neuroplasticity^69,70^. It is also interesting that studies using L-DOPA-carbidopa intestinal gel treatment have reported a benefit on both motor complications and ICBs^131,132^, indicating that not only the medication type but also the administration mode has a strong impact on the risk for impulse control disorders.

Furthermore, our results demonstrate that partial DA-depletion does not confer a greater risk of developing ICBs, indicating that D2/3 receptor stimulation is sufficient to induce these behaviours even with an intact dopaminergic system. Therefore, caution should be used when using D2/3 agonists in patients with conditions other than PD, such as restless legs syndrome or fibromyalgia, as they also are at risk of developing ICB under this pharmacotherapy^133–135^.

Finally, our study presents novel methodological options to examine ICB-related features that are induced by dopaminergic therapies. The elegant operant tests currently used to investigate ICBs in rodents are essential to deepen our understanding of different functional aspects contributing to these disorders (reviewed in refs. ^5,16,136^). However, they require long animal training periods with specific contingencies, which limit their throughput. The compulsive checking test and the behavioural segmentation analysis performed in this study do not require lengthy animal pre-training, operant contingencies, or food deprivation, thus being relatively simple to apply in most laboratory settings. Our results show that these tests enable the identification of a behavioural structure associated with an impulsive-compulsive phenotype. These methods can therefore be used to evaluate candidate treatments for D2/3 agonist-induced ICBs in translationally oriented investigations in the future.

## Methods

### Animals

Adult female Sprague-Dawley rats (Janvier Labs, France) of two months of age (weighing 250 g on arrival) were housed in Innovive cages (San Diego, CA, USA) on a 12:12 h light/dark cycle with ad libitum access to food and water unless otherwise stated. All procedures were approved by the Malmö-Lund Ethical Committee on Animal Research.

### Study design

Rats with bilateral striatal 6-OHDA lesions or sham lesions were allocated to four groups to receive the following treatments (administered as daily s.c. injections): (1) physiological saline solution (n_6-OHDA_ = 6 and n_sham_ = 7); (2) L-DOPA 24 mg/kg (LD24, n_6-OHDA_ = 10 and n_sham_ = 9); (3) the D2/3 receptor agonist ropinirole 2.5 mg/kg (R2.5, n_6-OHDA_ = 10 and n_sham_ = 9); (4) the combination of L-DOPA and ropinirole (LD24 + R2.5, n_6-OHDA_ = 10 and n_sham_ = 9). Injections were administered for five consecutive days (day 1 to day 5) with a drug-free period of two days (days 6 and 7) for a total of 6 weeks.

Behavioural assessments were carried out according to the timeline presented in Fig. 1a. Specifically, rats were evaluated in the open field test during treatment week 2, and in the EPM and compulsive checking test during week 3. Thereafter, all rats were video recorded for an analysis of active behaviours in a home cage-like environment. Then, the 6-OHDA-lesioned cohort continued in the rIGT protocol for the last three weeks of chronic treatment. After completing the behavioural test schedule, animals were euthanised one hour after their last s.c. injection and brains were collected for immunohistochemical analyses. One 6-OHDA-lesioned animal treated with ropinirole was excluded from these analyses due to technical issues in brain tissue preparation.

### Drug treatments

L-DOPA (L-3,4-dihydroxyphenylalanine methyl ester hydrochloride, Sigma Aldrich AB, Sweden) and ropinirole (cat# HB1923, HelloBio, UK) were dissolved in physiological saline solution (vehicle) and administered s.c. in a volume of 1 ml/kg. L-DOPA was coadministered with a fixed dose of a peripheral DOPA decarboxylase inhibitor (12 mg/kg, s.c. Benserazide hydrochloride, Sigma Aldrich AB, Sweden), which was mixed in the same saline solution. For the combined treatment, ropinirole was always prepared separately and injected simultaneously with L-DOPA. Drugs were injected 15 min before the beginning of each behavioural test. The drug doses used for chronic treatment were selected based both on literature review and pilot data with acute drug challenges, performed in a separate group of bilaterally 6-OHDA-lesioned rats (Fig. S4). The chosen drug doses were well tolerated, did not visibly alter any qualitative aspects of the rats’ behaviour, nor did they elicit any dyskinetic or dystonic features.

### Bilateral dopamine-denervating lesions

Bilateral DA denervation was performed by injecting the neurotoxin 6-OHDA into the dorsolateral striatum of both hemispheres according to published procedures^20,25^. Briefly, rats were anaesthetised with a single intraperitoneal (i.p.) injection of fentanyl/medetomidine (50 µg/ml and 1 mg/ml i.p., respectively; Apoteksbolaget) at a ratio of 1:20 and mounted in a stereotaxic frame (David Kopf Instruments, CA, USA). 6-OHDA hydrochloride (Sigma Aldrich AB, Sweden) was dissolved in 0.2% ascorbate-saline at a concentration of 3.75 µg/µl (free base) and injected into the dorsolateral striatum at the following coordinates (in mm relative to bregma and dural surface): AP + 0.7, ML ±3.4, DV −4.5, with tooth bar in flat skull position (approximately −4.5). A total of 2 µl toxin solution was injected at a rate of 0.5 µl/min using a 5 µl Hamilton syringe. The needle was left in place for 2 min after each injection. Sham lesions were carried out by inserting the needle at the above coordinates without carrying out the injection. Following surgery, the effect of anaesthesia was reversed by atipamezole hydrochloride (0.1 mg/kg, s.c.; Apoteket AB, Sweden), and animals received analgesic treatment with buprenorphine (0.01 mg/kg, s.c.; Apoteket AB, Sweden). The animals were kept under thorough observation for approximately two to three days, and given calory-enriched food (DietGel Boost, Clearh20.com) to facilitate their prompt recovery.

### Stepping test

Approximately three weeks after surgery, the efficacy of 6-OHDA lesions was evaluated by assessing forelimb akinesia in the stepping test. The ability of each rat to perform adjusting steps in response to experimenter-imposed lateral movements was investigated as previously described^20,48^. Briefly, the rat was held firmly by an experimenter lifting its hindlimbs and one forelimb, letting the unrestrained forelimb contact a table surface. Rats were moved 90 cm laterally during a 5 s period in the direction towards the rat’s midline, then back to the start point. A second experimenter counted the number of adjusting steps performed by the animal in both the forehand and backhand direction. The trial was repeated two times per forelimb. Three weeks post-lesion, animals were habituated to the handling associated with this test for three consecutive days, followed by one day of testing. The test was repeated after three weeks of drug treatment using the same protocol. On this occasion, the test was carried out at 25 min following drug injection to ensure comparable levels of motor activity after L-DOPA and ropinirole treatment. Results from this test are expressed as the number of adjusting forelimb steps performed with both paws, averaged across trials (Fig. 1b).

### Open field test

The open field test was performed to assess unconstrained motion patterns and anxiety-like behaviour, as previously described^137–139^. Animals were placed individually in the centre of a square arena (80 × 80 cm) surrounded by grey plastic walls. Animals were recorded for 60 min after a 30 min habituation period using a video camera suspended 2 m above the arena, and videos were analysed using the ANY-maze video tracking system (ANY-maze, Stoelting Europe, Ireland). Movements were tracked in different zones of the arena defined by the software. The 20% of the bottom surface closest to the walls were defined as the outer zone and the remaining 80% as the inner zone. The following measures were assessed: (i) Total distance travelled (m); (ii) Total time mobile (s); (iii) Maximal speed (m/s); (iv) Fraction of time spent in the inner zone (% of total recorded time).

### Elevated plus maze (EPM)

The EPM test was used to assess anxiety-related behaviours^140–142^ in a test apparatus consisting of two opposing open arms (50.8 cm long, 10.2 cm wide) and two opposing wall-enclosed arms (50.8 cm long, 10.2 cm wide, wall height 40.5 cm) mounted around an open, squared centre area (10.2 cm per side) (Fig. 3a, Med-Associates Inc., USA). The test chamber was mounted 75 cm above the floor. Each rat was placed individually in the centre at the beginning of the test and was allowed to explore the arena freely for 5 min. The test was carried out 15 min following drug injection. Behaviour was recorded by a video camera suspended 1.5 m above the arena and analysed using the ANY-maze video tracking system (ANY-maze, Stoelting Europe, Ireland). Movements were tracked in different zones of the arena defined in the software program. The following measures were assessed: (i) Arena occupancy as a heat map (s); (ii) Fraction of open arm entries (% of total arm entries); (iii) Total distance travelled (m).

### Analysis of active behaviours

Animals were placed individually in an empty, transparent home cage and habituated to the novel environment for 60 min. The following day, animals were recorded using a video camera placed at a fixed distance (20 cm) in front of the cage (Hero 4 https://gopro.com/en/us/update/hero4). Animals were individually filmed for one minute every 20 min for a total of one hour after administering the respective treatment. The behavioural analysis was carried out using the first 50 s of recording from each monitoring period. Video analysis was performed with the event tracker software JWatcher^143^. Each scored behavioural category was defined as a state, and their diversity was mainly based on A.E. Kelley^144^. Our list of states was customised to capture the entire range of behaviours expressed by the rats (locomoting, rearing, grooming, head bobbing, sniffing up and sniffing down). Sniffing behaviour was divided in the two categories “sniffing up” and “sniffing down” according to the original classification of these stereotypic behaviours provided by A.E. Kelley^144^.

The software allowed us to manually annotate the beginning and end of behavioural bouts in each state. Only unequivocally classifiable bouts were scored, excluding frames where the animal was facing away from the camera in a way that precluded a correct classification. If two to three types of behaviour were occurring simultaneously, the most prominent one (as for time duration) was annotated. A state analysis was performed by JWatcher, including the total time spent for each state (extension file: cd.res) and the given time at which every behaviour was scored (extension file: tr.res). The latter was used to calculate the duration of each behavioural bout and the number of switches between bouts. The analysis of time spent in each bout was performed only for the states exhibited by all the experimental groups (locomoting, rearing, sniffing up, and sniffing down). For the final analysis, the total time spent in active behaviours, the time per behavioural bout and the number of switches between states were averaged between 20, 40 and 60 min. One sham-lesioned animal under vehicle treatment was excluded from the analysis due to showing physical discomfort on the experiment day.

### Compulsive checking

Compulsive checking behaviour was assessed using a test originally introduced by Szechtman, et al.^27^. Briefly, 15 min following drug injection, animals were placed individually in the centre of a custom-made open square platform (140 × 140 cm) mounted 30 cm above the floor. Four plexiglass boxes open on one side (1 black box 8 × 8 × 8 cm, two transparent boxes 8 × 8 × 8 cm, 1 transparent box 8 × 8 × 11 cm), were placed at fixed locations (two near the middle and two near the corners of the platform, Fig. 5a). Animals were allowed to freely explore the arena for 45 min. Data was analysed using the complete 45 min period for vehicle and L-DOPA-treated rats, and using the last 30 min for hyperlocomotive rats (R2.5 and LD24 + R2.5 groups) as in previous studies^27,145^. Since D2 class agonists show biphasic effects on spontaneous locomotor behaviour^146^, we excluded the first 15 min of the observation period for behavioural assessment of rats receiving ropinirole treatment, either alone or combined with L-DOPA.

The behaviour was recorded by a video camera suspended 2 m above the arena and analysed using the ANY-maze video tracking system (ANY-maze, Stoelting Europe, Ireland). Movements were tracked in different zones of the arena defined in the software. More specifically, the platform was subdivided into 25 zones of 35 × 35 cm, of which the outer zone extended 17.5 cm outside the open field (Fig. 5a). According to Eilam and Golani^83^, rats establish one or two preferred locations (“home bases”) and organise their behaviour in relation to them. For each rat, we identified one or two non-adjacent home bases as the zones with the highest total duration of stopping, where stops refer to periods of no locomotion (immobile for >1 s)^27^. Home base checking is defined as the animal entering these locales, and compulsive checking behaviour is present if the rat returns to the home bases excessively often and rapidly, and visits fewer places before returning to the home bases^27^. Therefore, compulsive checking parameters were characterised with reference to the home bases and included: (i) Frequency of checking: total number of visits to the home bases; (ii) Ratio of observed to expected checks: home base visits normalised for changes in locomotive behaviour by calculating the ratio of observed home base visits to the average expected visits to any zone^27,105^; (iii) Mean visit time at home bases; (iv) Mean return time to home bases from other zones; (v) Average amount of stops between checks.

### Rat Iowa gambling task (rIGT)

In 6-OHDA-lesioned rats, decision-making with uncertain outcomes was investigated using the rIGT protocol introduced by van den Bos et al.^39^. Briefly, the rIGT apparatus consisted of a start box, a choice area, and four goal arms (Fig. 6a, for details see van den Bos et al.^39^). Before initiating the test protocol, rats were given one habituation session being able to freely explore the apparatus for 10 min. Two days later, they were mildly food deprived (90–95% free-feeding body weight) and performed a total of 120 trials across a period of 10 days (two 5-day periods on weekdays; food available on weekend days up to 100% of free-feeding consumption measures). At the beginning of each trial, animals were placed individually in the start box. Once the rat had entered a goal arm with its full body, the chosen arm was closed, preventing the rat from leaving the choice arm before exploring the reward cup (for details see van den Bos et al.^39^; de Visser et al.^116^).

Of the four goal arms, two were baited, and two were empty. The latter were introduced as a control for non-specific exploration and potential spatial learning deficits^28,116^. Of the two baited arms, the long-term advantageous one contained 1 reward pellet on 8 out of 10 trials (45 mg purified rodent pellets, 1811155 5TUL, Testdiet, USA, Missouri, Saint Louis) and a bitter, quinine-saturated pellet on the other two trials, thus delivering 8 pellets win per 10 choices. The long-term disadvantageous arm contained 3 reward pellets on 1 out of 10 trials, and quinine-saturated pellets on the remaining 9 trials (3 pellets win per 10 choices). The positions of baited and empty arms, as well as the advantageous and disadvantageous arms, were counterbalanced across rats to avoid bias in the experimental design. To help the rats differentiate the goal arms, different visual cues (10 × 10 cm; circle or cross drawing in white or black) were displayed on the walls near the entrance of each arm.

One week before the first rIGT session, animals were habituated to reward pellets and the reward cup in their home cage followed by a single session of providing pellets in a novel, empty cage similar to the home cage. All rats consumed the pellets. During the rIGT testing, most rats briefly tasted the quinine-saturated pellets and left them uneaten thereafter. Based on van den Bos et al.^39^, animals were excluded on any of the following conditions: (1) the rat did not leave the start box; (2) the rat did not make a choice while being in the choice area; (3) the rats ate quinine pellets. Furthermore, rats showing strong quinine-specific responses, such as drooling^147^, were excluded from the study. Because of the above criteria, *n* = 4 from LD24, *n* = 4 from R2.5, and *n* = 3 from LD24 + R2.5 groups were excluded from the test.

Groups were compared on the following measures: (i) number of empty arm visits as a fraction of the total number of visits; (ii) number of advantageous arm visits as a fraction of the total number of visits. Data were expressed in blocks of 12 trials, and scores from the last session were taken as a measure of final performance.

### Tissue preparation and immunohistochemistry

After six weeks of chronic treatment, animals were anaesthetised with a lethal dose of sodium pentobarbital (240 mg/kg i.p., Apoteksbolaget AB, Sweden) and transcardially perfused with 0.9% saline followed by 4% ice-cold buffered paraformaldehyde (VWR) 1 h following the last s.c. injection of drug(s) or saline. Brains were then rapidly extracted, post-fixed in the paraformaldehyde solution for 2 h, and transferred to 25% sucrose in 0.1 M phosphate buffer at 4 °C for cryoprotection. Coronal sections of 40 µm thickness were cut serially through the brain using a freezing microtome and stored in antifreeze solution (0.5 M sodium phosphate buffer, 30% glycerol, and 30% ethylene glycol) at −20 °C until further analysis.

For quantifying the extent of striatal dopaminergic denervation and counting of pS6^+^ cells, bright-field immunohistochemistry was performed using a primary antibody against TH (rabbit anti-TH, Pel-Freez P40101, 1:1000) and phosphorylated pS6, respectively (monoclonal rabbit anti Ser235/236-phospho-S6, Cell Signaling #2211, 1:200). Immunocomplexes were revealed using biotinylated secondary antibodies from Vector Laboratories (goat anti-rabbit BA 1000, 1:200 for TH and 1:400 for phosphorylated pS6), followed by avidin-biotin peroxidase solution (ABC Elite Kit, Vector Laboratories). The final colour reaction was developed using 3,3’-diaminobenzidine (DAB) in 0.04% H_2_O_2_. After all histochemical procedures, sections were mounted onto chrome alum-coated glass slides, dehydrated, and cover slipped using D.P.X. mounting medium (Sigma Aldrich).

### Quantitative image analysis

Quantitative analysis of striatal TH immunostaining was carried out in a blinded fashion to verify the lesion extent. Images were acquired from the main body of the caudate-putamen using a Nikon 80i microscope connected with a digital camera (Olympus DP72). They were scanned under a 4x objective and merged into a single image covering the entire striatum. Three rostrocaudal levels were selected for the quantification (AP + 1.08, +0,72, and +0,12 mm relative to bregma, according to Paxinos and Watson^148^). A manual tracing tool (Fiji, ImageJ2 v2.8.0, NIH) was used to outline both the entire caudate-putamen (“total area”) and the region lacking TH immunoreactivity (“lesioned area”), as in Wang et al.^149^. Data were expressed as the percentage of the DA-intact area of the total area measured.

For the count of TH-positive neurons in the substantia nigra pars compacta, three identical levels along the anterior-posterior axis were selected in each animal (corresponding to Bregma −5.8, −6.00 and −6.2 in the rat brain atlas by Paxinos and Watson^148^). These levels were chosen as they enable to readily distinguish the compacta cell group medial to the ventral tegmental area from the central and lateral portions of the substantia nigra pars compacta, which host DA neurons innervating the dorsolateral striatum^150^. A Nikon 80i microscope connected with a digital camera (Olympus DP72), supporting an x-y motorised stage guided by NewCAST software, was used for the analysis (version 4.4.4.0, Visiopharm, DK; see Westin et al.^151^ for additional details). The ventrolateral portion of the substantia nigra pars compacta, where dopaminergic neurons projecting to the dorsolateral part of the striatum originate, was defined as the region of interest (ROI), thus bypassing the medial part converging with the ventral tegmental area (VTA) or the parabrachial pigmented nucleus and the medial tier of the substantia nigra pars compacta.

ROIs were acquired under a 4X objective for both hemispheres and then randomly sampled with the 40X objective by the NewCAST software to cover 100% of the ROI. The total number of TH-positive cells was calculated by summing up the average number of cells counted on both sides for each of the three levels in a sample of sham (*n* = 9) and all 6-OHDA animals (*n* = 36). Data were expressed as a percentage of TH-positive cells in the 6-OHDA-lesioned animals compared with the average of TH-positive cells in sham-lesioned animals.

For quantifying pS6^+^ cells, sections through the same rostrocaudal levels used for TH quantification were scanned under a 20X objective using a Hamamatsu S210 microscope (LRI Olympus, Sweden). The total striatal area and lesioned area were outlined manually after overlaying the TH-immunostained sections onto the ones stained for phosphorylated pS6. The software ImageJ (version 1.54t, National Institute of Health, USA) was used to both count pS6^+^ cells and determine their XY coordinates and outlines within the section. Cells were regarded as pS6-positive when their optical density exceeded the one measured in the surrounding neuropile by at least 3-fold. Cell counts were expressed as the number of pS6^+^ cells per unit area (mm^2^), averaged bilaterally over the three rostrocaudal levels in each animal, and averaged across the denervated areas in 6-OHDA-lesioned animals.

### 2D histograms of striatal activity and principal component analysis

With the goal of mapping striatal activity, XY coordinates of pS6^+^ cells were imported into MATLAB (version R2022b) together with the striatal outlines traced in ImageJ. Coordinates were adjusted for each section to optimise the alignment of the outlines. These adjustments were done manually and allowed for rotation, isometric scaling, and translation. Right-side striatum coordinates were flipped horizontally and fitted to the left side. 2D histograms of cell counts with a bin size of 80 × 80 µm (75 × 75 pixels) were created for each section, obtaining a 2D image representing the distribution of pS6^+^ cells in the coronal plane. Differences in pS6^+^ cell counts between groups were calculated by subtracting the average cell count map of one experimental group from another one (Fig. S2, rows 1 and 3). Maps of statistically significant differences were created by pixelwise, univariate comparisons between the two groups (see rows 2 and 4 in Fig. S2 and Statistical analysis).

Additionally, PCA was used to identify the main patterns of covariation in pS6^+^ cell distributions^152,153^. To this end, a matrix was constructed where each row corresponded to the 2D histogram of one section and each column corresponded to one pixel (411 × 5625). The entire material (all rostrocaudal levels, animals and groups; n(independent animals)=69, n(sections) = 411) was used to perform one PCA (Matlab function pca.m). The 2D histogram for each striatal section, for example Section A, can be described by a linear equation based on all PCs and their corresponding coefficients for the respective section^153^:

1 $$\begin{array}{rcl}\begin{array}{l}2{\rm{D}}\,{\rm{h}}{\rm{i}}{\rm{s}}{\rm{t}}{\rm{o}}{\rm{g}}{\rm{r}}{\rm{a}}{\rm{m}}({\rm{S}}{\rm{e}}{\rm{c}}{\rm{t}}{\rm{i}}{\rm{o}}{\rm{n}}\,{\rm{A}})={{\rm{C}}{\rm{o}}{\rm{e}}{\rm{f}}{\rm{f}}{\rm{i}}{\rm{c}}{\rm{i}}{\rm{e}}{\rm{n}}{\rm{t}}}_{{\rm{P}}{\rm{C}}1}{\rm{A}}\,{\rm{x}}\,{\rm{P}}{\rm{C}}1\\ +{{\rm{C}}{\rm{o}}{\rm{e}}{\rm{f}}{\rm{f}}{\rm{i}}{\rm{c}}{\rm{i}}{\rm{e}}{\rm{n}}{\rm{t}}}_{{\rm{P}}{\rm{C}}2}{\rm{A}}\,{\rm{x}}\,{\rm{P}}{\rm{C}}2{+}_{\ldots }+{{\rm{C}}{\rm{o}}{\rm{e}}{\rm{f}}{\rm{f}}{\rm{i}}{\rm{c}}{\rm{i}}{\rm{e}}{\rm{n}}{\rm{t}}}_{{\rm{P}}{\rm{C}}410}{\rm{A}}\,{\rm{x}}\,{\rm{P}}{\rm{C}}410\end{array}\end{array}$$

Thus, the average coefficient for a PC in one experimental condition can be used as a measure of expression of the covariance pattern described by this PC. The first three PCs were chosen for further analyses as they explained a larger fraction of overall variance relative to any other PCs (PC1: 26%, PC2: 4%, PC3: 3%) and were plotted as patterns of covariance in pS6^+^ cell distribution (Fig. 8a–c). To quantify the expression of the corresponding covariance patterns, the first three PC coefficients were then compared between groups (Fig. 8a’–c’ and S3).

### Statistical analysis

Statistical analysis was carried out using RStudio (version 2022.12.0) and GraphPad Prism (version 10.2.2). Assumptions of normality and homoscedasticity were analysed graphically using QQ and residual plots. Data were analysed using two-factor ANOVAs or mixed effect models where appropriate, followed by Tukey’s multiple comparison test for post hoc comparisons. For data that were not normally distributed, a Kruskal–Wallis test and post hoc Dunn’s test were used. Pairwise comparisons of treatment effects were primarily performed within each lesion type (sham or 6-OHDA). When no significant overall effect of lesion type or lesion x treatment interaction was detected in the two-factor ANOVA, post hoc comparisons of treatment effects were carried out combining sham and 6-OHDA groups in each treatment arm.

Mann-Whitney U tests were used to compare pS6^+^ cell counts in 2D histograms between two groups for each pixel, and multiplicity was accounted for by lowering the level of significance to α = 0.01 based on the Benjamini-Hochberg correction for the false discovery rate^154^. In all other analyses, the level of statistical significance was set to α = 0.05, and all tests were performed two-sided. Bar plots for parametric analyses display group mean ± SEM and boxplots for non-parametric analyses median and quartiles.

## Supplementary information

Supplementary material_revised_v4

Video 1 - SHAM Saline

Video 2 - SHAM LD24

Video 3 - SHAM R2.5

Video 4 - SHAM LD24+R2.5

Video 5 - 6-OHDA Saline

Video 6 - 6-OHDA LD24

Video 7 - 6-OHDA R2.5

Video 8 - 6-OHDA LD24+R2.5

## Data Availability

The datasets used and analysed during the current study are available from the corresponding author on reasonable request.

## Abbreviations

6-OHDA: 6-hydroxydopamine
DA: dopamine
EPM: elevated plus maze
i.p.: intraperitoneal
ICB: impulsive-compulsive behaviour *[line break]* ICD impulse control disorder
LD24: L-DOPA 24 mg/kg
LD24 + R2.5: combination of L-DOPA 24 kg/mg and ropinirole 2.5 mg/kg
PC: principal component
PCA: principal component analysis
PD: Parkinson’s disease
pS6: phosphorylated ribosomal protein S6
pS6^+^ cells: phosphorylated pS6-immunopositive cells
R2.5: ropinirole 2.5 mg/kg
rIGT: rat Iowa Gambling Task
s.c.: subcutaneous
TH: tyrosine hydroxylase

## Code availability

The code applied to obtain groupwise 2D histograms of pS6^+^ cells and to perform a PCA on main patterns of striatal neuroactivity is available at: https://github.com/NRC-Lund/paper-wolfschlag-2024.
