# Supplementary material for: Impulsive-compulsive behaviours and striatal neuroactivity in mildly parkinsonian rats under D2/3 agonist and L-DOPA treatment
Source: NPJ Parkinsons Dis. 2025 May 29;11:142. doi: 10.1038/s41531-025-00996-z (PMC12122685; doi:10.1038/s41531-025-00996-z)
Supplement: Supplementary file 1 — Supplementary material_revised_v4 [file 41531_2025_996_MOESM1_ESM.docx]

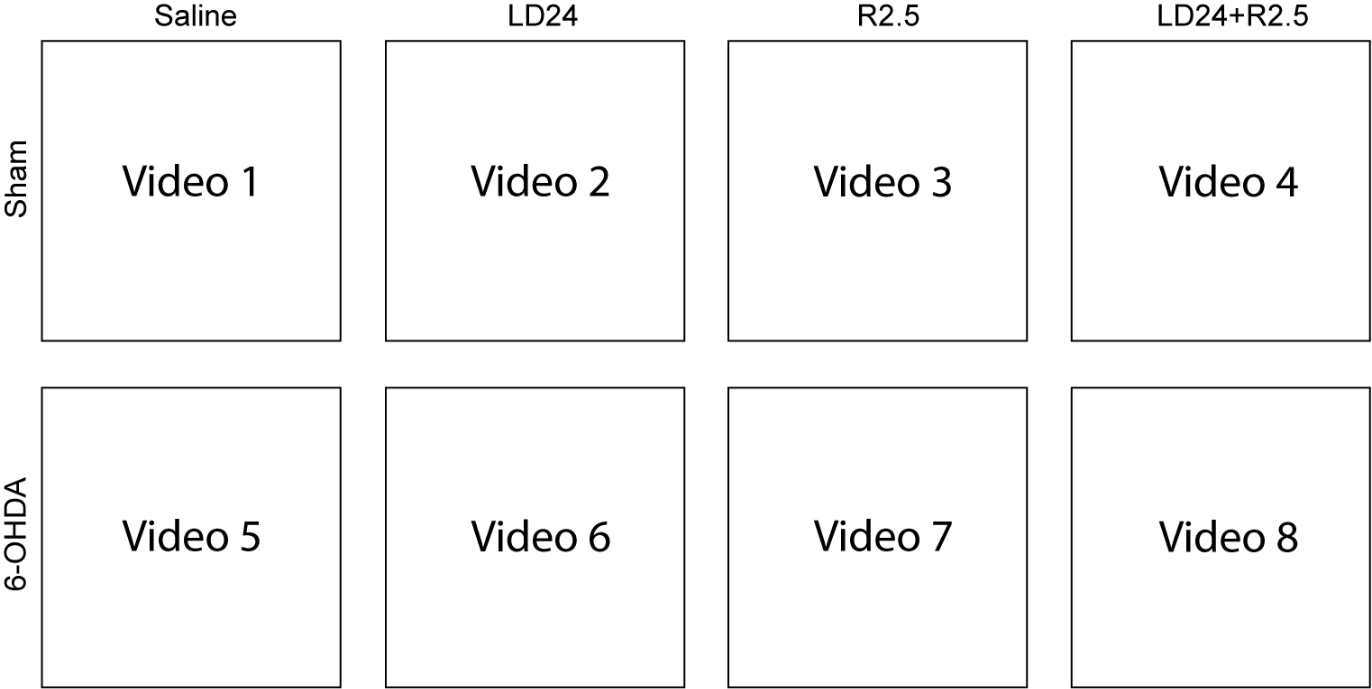


**Figure S1. Video clips of movements in the compulsive checking test.** Thirty-second representative movement patterns in the arena are shown in one example animal for each experimental group. Video 1-4: sham-lesioned rats treated with Saline (video 1), LD24 (video 2), R2.5 (video 3) and LD24+R2.5 (video 4). Video 5-8: 6-OHDA-lesioned rats treated with Saline (video 5), LD24 (video 6), R2.5 (video 7) and LD24+R2.5 (video 8). In the PDF version of this article, please click anywhere on the figure or caption to play the video in a separate window.

**Supplementary Video 1.** Thirty-second representative movement patterns of sham-lesioned rats under Saline treatment in the compulsive checking arena.

**Supplementary Video 2.** Thirty-second representative movement patterns of sham-lesioned rats under LD24 treatment in the compulsive checking arena.

**Supplementary Video 3.** Thirty-second representative movement patterns of sham-lesioned rats under R2.5 treatment in the compulsive checking arena.

**Supplementary Video 4.** Thirty-second representative movement patterns of sham-lesioned rats under LD24+R2.5 treatment in the compulsive checking arena.

**Supplementary Video 5.** Thirty-second representative movement patterns of 6-OHDA-lesioned rats under Saline treatment in the compulsive checking arena.

**Supplementary Video 6.** Thirty-second representative movement patterns of 6-OHDA-lesioned rats under LD24 treatment in the compulsive checking arena.

**Supplementary Video 7.** Thirty-second representative movement patterns of 6-OHDA-lesioned rats under R2.5 treatment in the compulsive checking arena.

**Supplementary Video 8.** Thirty-second representative movement patterns of 6-OHDA-lesioned rats under LD24+R2.5 treatment in the compulsive checking arena.


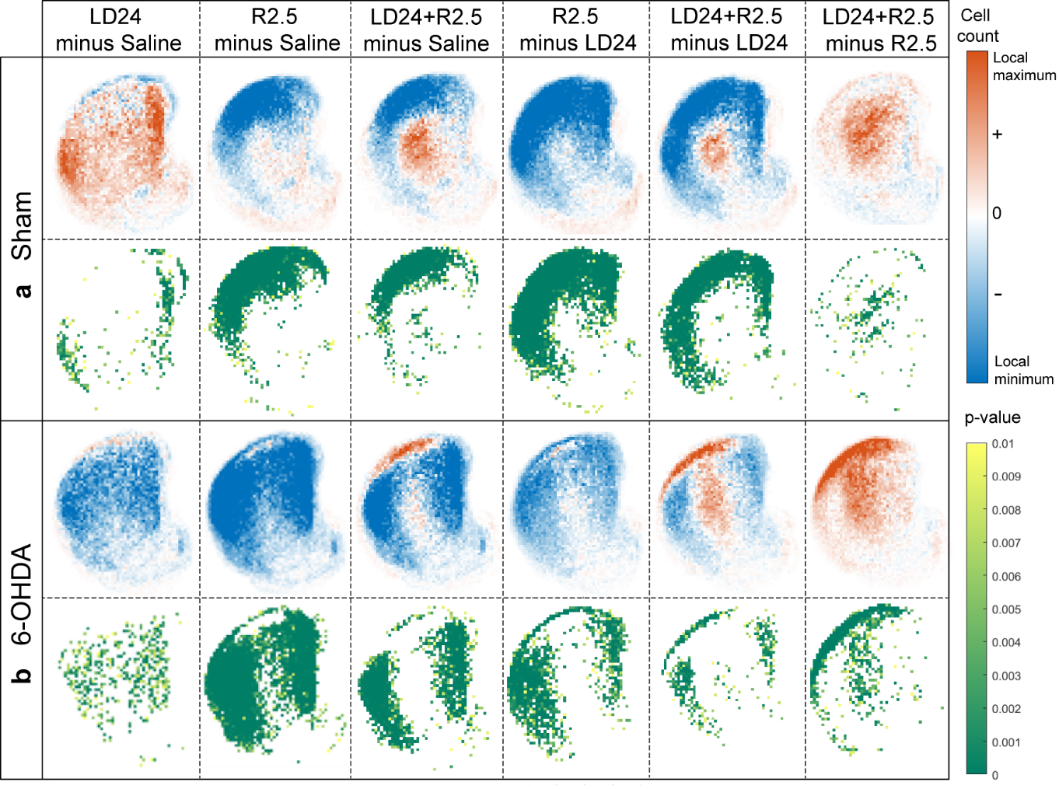


**Figure S2. Differences in pS6^+^ cell distribution between groups.** Blue-red images (row 1 and 3) show relative pS6^+^ cell counts (Group 1 minus Group 2). Green-yellow images (row 2 and 4) show statistical maps of p-values <0.01 in Mann-Whitney U tests between two corresponding unit areas. Image dimension: 75x75 pixel (80x80 μm). The significance threshold was adjusted to α=0.01 using the Benjamini-Hochberg method. n(independent animals)=69, n(striatal sections)=411. **(a)** Groupwise comparisons by subtraction between sham-lesioned animals under saline, LD24, R2.5, and LD24+R2.5 treatment. **(b)** Groupwise comparisons by subtraction between 6-OHDA-lesioned animals under saline, LD24, R2.5, and LD24+R2.5 treatment.


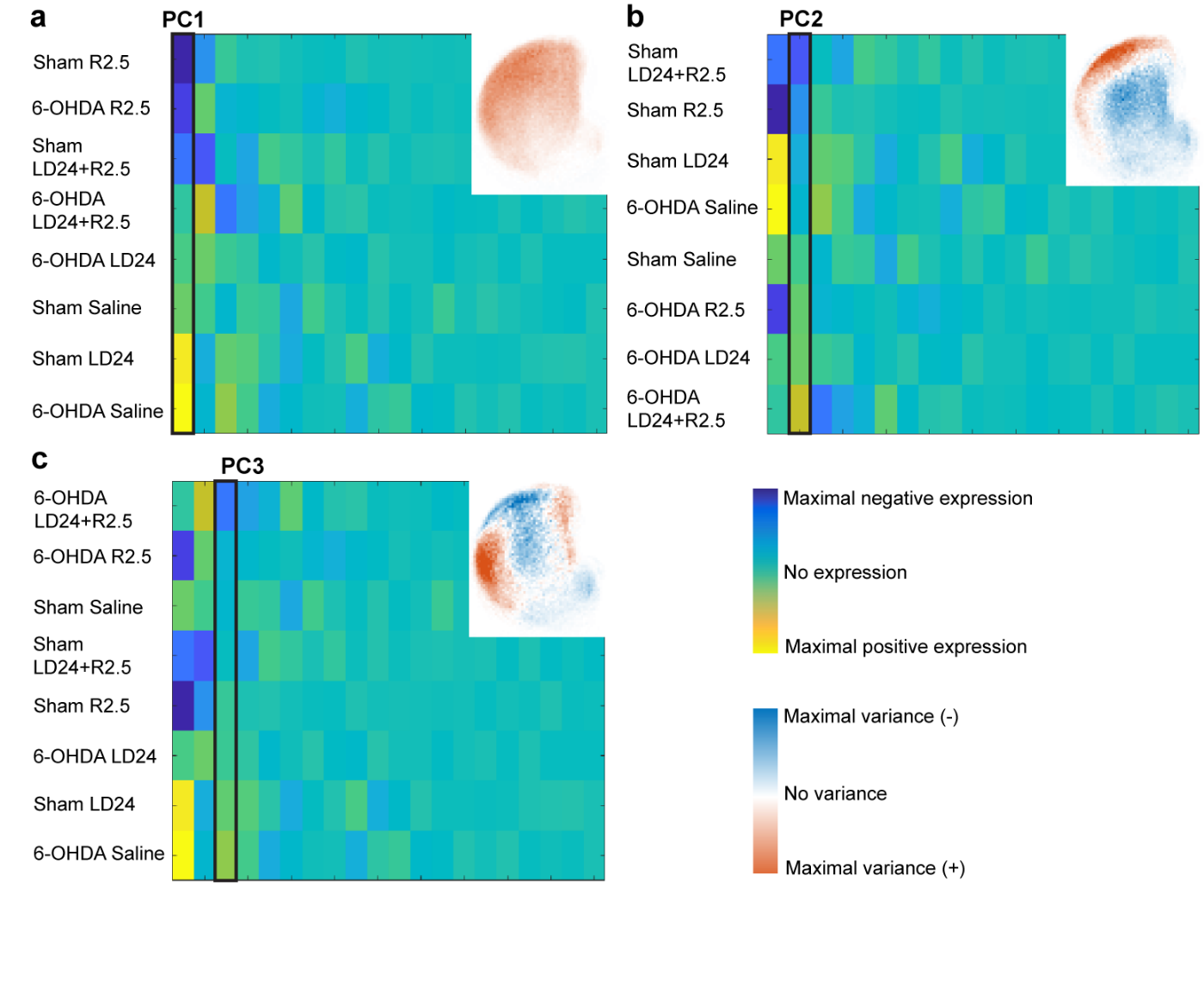


**Figure S3. Average expression of principal components (PCs) 1-20 in different treatment groups in sham- and 6-OHDA-lesioned animals.** n(independent animals)=69, n(sections)=411. **(a)** Sorted by ascending PC1 expression; image of PC1 in right upper corner. **(b)** Sorted by ascending PC2 expression; image of PC2 in right upper corner. **(c)** Sorted by ascending PC3 expression; image of PC3 in right upper corner.

**Acute-dose response experiment and choice of the drug doses**

In a pilot experiment, rats with bilateral 6-OHDA lesions were sequentially treated with saline, followed by ascending doses of ropinirole (0.5, 2.5, and 5.0 mg/kg s.c. as daily injection) and then L-DOPA (6, 12, 24, and 50 mg/kg s.c. as daily injection). Consecutive doses of the same drug were given at least 2 days apart, and 7 days were allowed between the two drug blocks. Animals were placed in the compulsive checking test apparatus (one rat at a time) and recorded for 45 min, starting 15 min after the s.c. injection (see Methods paragraph 5.9). During the test, animals were carefully observed to detect possible adverse motor effects of the treatments (including dyskinesia or dystonia of specific body parts).

The low and intermediate doses of ropinirole (R0.5, R2.5) and L-DOPA (LD6, LD12, LD24) did not visibly alter any physical or qualitative feature of the animals’ spontaneous behaviours. However, the highest dose of each drug (R5.0, LD50) appeared to impair the animals’ motor coordination during the execution of specific actions. This impairment was mainly evident when rats were climbing on the plastic boxes placed within the test apparatus, and they could not fully complete this action under the effect of the highest drug doses.

The results from the compulsive checking test are shown in Fig. S4. Overall, the effect of L-DOPA was minimal, and the only significant effect observed was an increase in the frequency of checking with the highest dose (Fig. S4a, p<0.05 vs LD6). No significant effects of any L-DOPA dose were observed on the other parameters (Fig. S4b-e). When compared to both L-DOPA and saline, all doses of ropinirole similarly increased the frequency of checking (Fig. S4a), the ratio between observed and expected checks (Fig. S4b), and the visit time at the home base (Fig. S4c). However, the two parameters return time to home base (Fig. S4d) and stops between checks (Fig. S4e) were reduced only by the highest dose of ropinirole (p<0.05 for R5.0 vs LD6), whereas the lower and intermediate doses (R0.5 and R2.5) did not reach a significant effect.


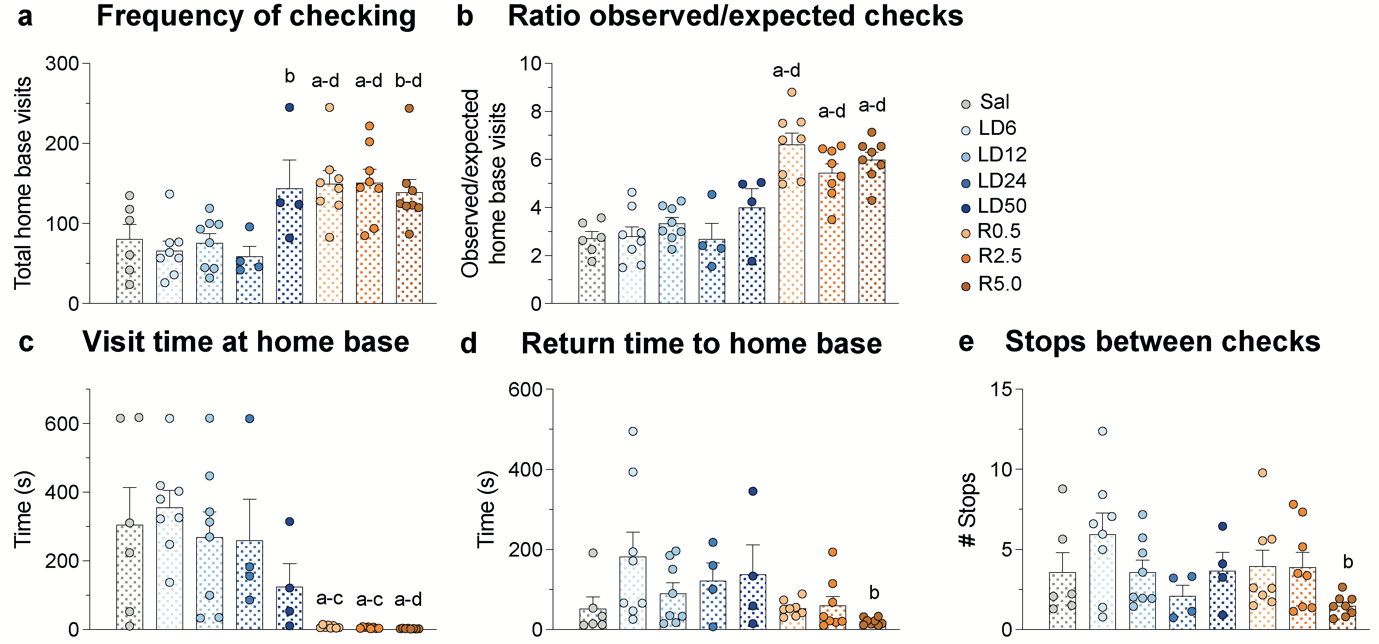


**Figure S4. Compulsive checking parameters in the initial acute-dose response study.** A pilot experiment was performed in 6-OHDA-lesioned animals (n=8) treated with saline and different acute doses of L-DOPA and ropinirole. The highest L-DOPA doses (LD24 and LD50) were tested in n=4 rats per dose, and saline in n=6. Data were analysed with repeated measurement Mixed effect models followed by Tukey’s post hoc test for the treatment effect. **(a)** Frequency of checking (total home base visits), F(treatment)_7,39_ = 8.4, p<0.001. **(b)** Ratio observed/expected home base visits, F(treatment)_7,39_ = 16.8, p<0.001. **(c)** Mean visit time at home base, F(treatment)_7,39_ = 9.4, p<0.001. **(d)** Mean return time to home base, F(treatment)_7,39_ = 3.0, p=0.011. **(e)** Average stops between checks, F(treatment)_7,39_ = 2.7, p=0.027. Symbols of statistical significance: a: p<0.05 vs Saline, b: p<0.05 vs LD6, c: p<0.05 vs LD12, d: p<0.05 vs LD24.

In summary, the results of this acute dose-response study show that, differently from L-DOPA, ropinirole induced features of compulsive checking at all doses tested. A ceiling effect was reached at the low and intermediate doses on certain parameters (in particular, frequency of checking, return time to home bases) but not on others (return time to home base, stops between checks). On the latter parameters, only the highest dose (R5.0) had a significant effect.

Previous studies on the effects of pramipexole in the delay discounting task have shown that this D3/D2 receptor agonist affected impulsive choices in a dose-dependent manner ^1,2^. Intrinsic differences between tasks are likely to underlie their different dose-dependency. The compulsive checking test evaluates the presence or absence of specific actions ^3^. In the delay discounting test, however, the dependent variable (e.g., proportion of choices for a larger, delayed reward) can vary more continuously on a wide scale, potentially offering a better possibility to observe graded, dose-dependent shifts below the ceiling (100% preference for one option).

In addition to the above data, the following considerations factored in upon selecting the drug doses for the chronic study: (i) the 24 mg/kg L-DOPA dose was consistent with previous studies in rats with partial dopaminergic lesions ^4,5^, (ii) the 2.5 mg/kg ropinirole dose was well aligned with the pramipexole dose (3.0 mg/kg) reported to induce a stronger appearance of ICB-like features in DA-denervated compared to intact rats using the same 6-OHDA lesion model as in our study ^2^. In the same study, a low dose of pramipexole (0.25 mg/kg) was either ineffective or affected only a few parameters of impulsivity. The comparison with pramipexole is relevant because both pramipexole and ropinirole are D2/3 agonists with low nanomolar affinity for D2 and D3 receptors ^6^.


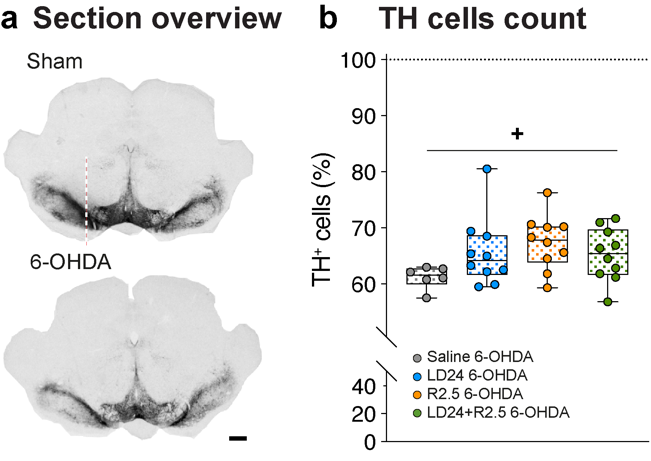


**Figure S5. Counts of TH-positive neurons in the substantia nigra. (a)** Representative overview of the substantia nigra pars compacta immunostained for tyrosine hydroxylase (TH) in bilaterally sham- (top) and 6-OHDA-lesioned rats (bottom). The dashed line in the sham section represents the anatomical border used to define the region of interest. Cells were counted laterally to this line, thus excluding the medial part of the substantia nigra pars compacta and the ventral tegmental area. Scale bar: 600 µm. (b) Data are expressed as the percentage of TH-positive cells in the 6-OHDA lesioned animals compared to the average of TH-positive cells in sham-lesioned animals, represented with the dashed line. (Kruskal-Wallis test: KW(treatment)=25.38 p<0.001). Symbols of statistical significance: + = p<0.05 vs. all Sham.

**References**

1 Holtz, N. A., Tedford, S. E., Persons, A. L., Grasso, S. A. & Napier, T. C. Pharmacologically distinct pramipexole-mediated akinesia vs. risk-taking in a rat model of Parkinson's disease. *Prog Neuropsychopharmacol Biol Psychiatry* **70**, 77-84, doi:10.1016/j.pnpbp.2016.05.004 (2016).

2 Jiménez-Urbieta, H. *et al.* Motor impulsivity and delay intolerance are elicited in a dose-dependent manner with a dopaminergic agonist in parkinsonian rats. *Psychopharmacology (Berl)* **237**, 2419-2431, doi:10.1007/s00213-020-05544-6 (2020).

3 Szechtman, H., Sulis, W. & Eilam, D. Quinpirole induces compulsive checking behavior in rats: a potential animal model of obsessive-compulsive disorder (OCD). *Behav Neurosci* **112**, 1475-1485, doi:10.1037//0735-7044.112.6.1475 (1998).

4 Carvalho, M. M. *et al.* Effect of Levodopa on Reward and Impulsivity in a Rat Model of Parkinson's Disease. *Front Behav Neurosci* **11**, 145, doi:10.3389/fnbeh.2017.00145 (2017).

5 Marin, C. *et al.* Early L-dopa, but not pramipexole, restores basal ganglia activity in partially 6-OHDA-lesioned rats. *Neurobiol Dis* **64**, 36-47, doi:10.1016/j.nbd.2013.12.009 (2014).

6 Coldwell, M. C., Boyfield, I., Brown, T., Hagan, J. J. & Middlemiss, D. N. Comparison of the functional potencies of ropinirole and other dopamine receptor agonists at human D2(long), D3 and D4.4 receptors expressed in Chinese hamster ovary cells. *Br J Pharmacol* **127**, 1696-1702, doi:10.1038/sj.bjp.0702673 (1999).
